# H4K12 Lactylation Impairs Dopaminergic Neuron Energy Metabolism via the HIF‐1α/NDUFS1 Pathway in Parkinson's Disease

**DOI:** 10.1002/advs.78220

**Published:** 2026-10-08

**Authors:** Yingyao Liu, Xi Li, Wenxuan He, Fenglin Li, Qican Ou, Yao Liu, Jiang Lei, Yanzi Bao, Huaxin Huang, Wei Feng, Yuanguang Tang, Xiaohui Qiu, Lu Gan, Yuefei Shen, Jin Wang, Yousheng Xiao

**Affiliations:** ^1^ Department of Neurology the First Affiliated Hospital of Guangxi Medical University Nanning China; ^2^ Department of Neurology Wuming Hospital Affiliated to Guangxi Medical University Nanning China; ^3^ Department of Neurology Guangxi Hospital Division of The First Affitiated Hospital Sun Yat‐sen University Nanning China

**Keywords:** energy metabolism, H4K12 lactylation, HIF‐1α, NDUFS1, Parkinson's disease

## Abstract

Parkinson's disease (PD) is a progressive neurodegenerative disorder characterized by the loss of dopaminergic (DA) neurons in the substantia nigra, with mitochondrial dysfunction central to its pathology. Recent studies have linked lactate—accumulating in PD due to mitochondrial dysfunction—to disease mechanisms through lactylation, a novel post‐translational modification. However, the specific pathogenic role of lactylation in PD remains unclear. Here, H4K12 was identified as a key histone lactylation site and its role in PD pathogenesis was investigated. Plasma lactate and global histone lactylation were elevated in 83 PD patients compared with 83 Health controls. Consistently, lactate and H4K12la were upregulated in both PD mouse and cell models. Genome‐wide CUT&Tag and RNA sequencing revealed that H4K12la enrichment at the HIF‐1α promoter upregulates HIF‐1α, which in turn represses *NDUFS1* via direct promoter binding, impairing mitochondrial respiration and disrupted cellular energy supply. Upstream, P300 was identified as a critical catalyst of H4K12la; P300 directly bound to and sustained H4K12la levels. Collectively, these findings delineate a pathogenic signaling axis—the P300/H4K12la/HIF‐1α/NDUFS1 axis—that disrupts energy homeostasis in DA neurons. Importantly, the pharmacological inhibition of this axis with 2‐deoxy‐D‐glucose or PX‐478 rescued motor deficits in PD model mice, thereby highlighting its therapeutic potential for PD.

## Graphical abstract

Lactate and H4K12la are elevated in Parkinson's disease. Mechanistically, p300‐mediated H4K12la enrichment at the HIF‐1α promoter upregulates HIF‐1α, which represses Ndufs1, impairing mitochondrial respiration and energy homeostasis in DA neurons. Pharmacologically targeting this axis with 2‐deoxy‐D‐glucose or PX‐478 rescues motor deficits, highlighting therapeutic potential.

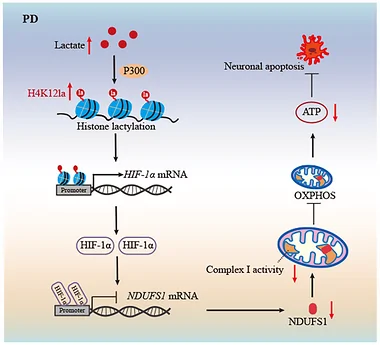

## 1 Introduction

Parkinson's disease (PD) is a common neurodegenerative disorder that is primarily characterized by progressive motor dysfunction. Its core pathological feature is the selective loss of dopaminergic (DA) neurons in the substantia nigra, which leads to a severe depletion of striatal dopamine [1]. Although the precise etiology remains poorly understood, PD pathogenesis is closely associated with mitochondrial dysfunction, oxidative stress, neuroinflammation, and abnormal protein aggregation [2]. Metabolic dysregulation has emerged as a critical component within this pathological landscape. Lactate—the end product of glycolysis—serves as a key metabolic indicator. In the brain, the L‐isomer of lactate predominates [3]. Under conditions of energy stress or hypoxia, pyruvate generated from glycolysis is converted to lactate by lactate dehydrogenase A (LDHA) [4]. Notably, magnetic resonance spectroscopy (MRS) studies have detected elevated lactate levels in the brains of PD patients, suggesting a pathological accumulation of this metabolite [5]. Moreover, in PD patients, the activity of mitochondrial complex I (CI) is markedly reduced in the substantia nigra. This CI dysfunction likely impairs oxidative phosphorylation in the electron transport chain (ETC), thus compromising ATP production and consequently driving cells toward glycolytic ATP generation. This metabolic shift results in excessive lactate production and its subsequent accumulation in brain tissue [6]. However, beyond its role as a metabolic byproduct, the specific mechanisms through which lactate contributes to neuronal vulnerability in PD are not well defined.

In recent years, epigenetic modifications—especially protein modifications that regulate gene expression and affect cellular function at the molecular level—have gradually attracted attention in neurodegenerative diseases [7]. Of these, protein lactylation, which is a novel epigenetic mark in which a lactyl group is covalently conjugated to lysine residues, has garnered considerable attention for its direct link to cellular metabolism. The levels of this modification are dynamically regulated by intracellular and extracellular lactate concentrations [8]. Protein lactylation modification can be classified into histone lactylation and non‐histone lactylation based on the protein substrate. The process is mediated by specific “writer” enzymes (e.g., histone acetyltransferase P300/CBP, histone acetyltransferase GCN5, histone acetyltransferase KAT7/HBO1) and “eraser” enzymes (e.g., histone deacetylase 1–3, NAD‐dependent protein deacetylase sirtuin‐1–3), thereby constituting an enzyme‐catalyzed regulatory axis, although non‐enzymatic lactylation also occurs [9, 10]. Histone lactylation directly promotes gene transcription and participates in diverse biological processes including tumor progression, inflammation, angiogenesis, and macrophage polarization [11, 12, 13]. Beyond its roles in cancer and immunometabolism, histone lactylation plays distinct roles in the nervous system. For example, following cerebral ischemia, elevated lactate levels promote histone H3K18 lactylation, which then induces neuronal apoptosis and exacerbates brain injury [14]. In Alzheimer's disease, H4K12 lactylation (H4K12la) enriches at glycolytic gene promoters in microglia, thus enhancing hypoxia‐inducible factor‐1α (HIF‐1α), pyruvate kinase PKM2, and LDHA expression to form a feedforward loop that aggravates neuroinflammation and cognitive decline [15]. Similarly, in schizophrenia models, enhanced glycolysis and lactate accumulation increase H3K9la, H3K18la, and high mobility group protein B1 expression, thereby promoting hippocampal neuronal apoptosis, whereas glycolysis inhibition alleviates behavioral deficits [16]. Furthermore, in glioblastoma, lactate‐induced H3K18la upregulates CD39, CD73, and C‐C chemokine receptor type 8, thus fostering an immunosuppressive microenvironment, and also promotes vasculogenic mimicry and tumor stemness [17, 18]. In PD, H3K9la transcriptionally activates solute carrier family 7 member 11, thereby promoting microglial activation and mediating neuroinflammation [19]. However, the pathological mechanisms by which histone lactylation mediates dopaminergic neuronal damage and loss in PD remain unclear.

In the present study, we first identified elevated cerebral lactate in PD patients vs. healthy controls (HCs) using multinuclear MRS, which revealed a significantly higher incidence of lactate peaks in the striatum of PD patients. Consistent with this observation, serum lactate levels were markedly increased in the PD cohort. These findings suggest that lactate accumulation may serve as an early biomarker of mitochondrial dysfunction and might be a key driver of pathogenic protein lactylation in PD. To investigate how lactate‐driven lactylation might contribute to dopaminergic neuron failure, we integrated multiple experimental approaches, including CUT&Tag, chromatin immunoprecipitation‐quantitative polymerase chain reaction (qPCR), RNA sequencing (RNA‐seq), and dual‐luciferase reporter assays. Our results delineate a coherent pathogenic axis by which lactate promotes P300‐mediated H4K12la, thereby leading to the transcriptional activation of HIF‐1α and the subsequent repression of the mitochondrial CI subunit NADH‐ubiquinone oxidoreductase 75 kDa subunit (NDUFS1). This lactate/P300/H4K12la/HIF‐1α/NDUFS1 cascade may disrupt energy homeostasis in dopaminergic neurons and exacerbate mitochondrial dysfunction. Targeting this axis might offer a novel therapeutic strategy for PD.

## 2 Materials and Methods

### 2.1 Participants

This study enrolled 83 patients with PD diagnosed according to the Movement Disorder Society (MDS) clinical diagnostic criteria, along with 83 age‐ and sex‐matched healthy controls (HCs) without motor or cognitive impairments, from the Department of Neurology at the First Affiliated Hospital of Guangxi Medical University. Participants with a history of diabetic ketoacidosis, hepatic or renal diseases, tumors, epilepsy, pancreatitis, or infectious diseases were excluded [20, 21, 22]. All participants were recruited between February 2024 and December 2025. Written informed consent was obtained from each participant prior to inclusion. The study protocol was approved by the Medical Ethics Committee of the First Affiliated Hospital of Guangxi Medical University (Approval No. 2024‐S125‐01). Randomization and blinding were not applicable to the observational design. The sample size was determined based on parameters reported in comparable published studies [23].

### 2.2 Clinical Assessment

Baseline demographic and clinical data, including sex, age, age of onset, disease duration, history of smoking and alcohol consumption, and medication use, were collected from all participants. Fasting venous blood samples were collected under resting conditions (after ≥15 min of seated rest). Plasma lactate levels were measured using the lactate oxidase method (reference range: 0.6–2.22 mmol/L). Disease severity in PD participants was assessed with the Hoehn and Yahr (H&Y) staging system. Motor symptoms were evaluated using Part III of the Unified Parkinson's Disease Rating Scale (UPDRS‐III), and medication intensity was quantified with the Levodopa Equivalent Daily Dose (LEDD) [24].

Participants who were able to complete a standardized lactate exercise test underwent additional blood sampling immediately after exercise and following a 10 min recovery period. Subjects performed moderate intensity stair climbing exercise for 5 min, ascending and descending a three‐story building at a speed corresponding to 40% – 50% of their individual maximum speed [25]. Individuals with muscle strength of Grade 4 or less, as well as those unable to climb stairs, were excluded from the study. Participants who completed fewer than three or more than four stair cycles within the 5 min period were asked to repeat the test on a separate day. Magnetic resonance spectroscopy (MRS) of the bilateral striatum was performed using a 3.0‐T MRI system at the Radiology Department of the First Affiliated Hospital of Guangxi Medical University for participants without contraindications. Presence of lactate peaks in the striatal regions of interest (ROIs) was documented.

### 2.3 Blood Sample Collection and Purification

Following clinical evaluation, venous blood samples were obtained from participants using heparinized vacuum tubes. Peripheral blood mononuclear cells (PBMCs) were isolated by density gradient centrifugation. PBMCs were lysed using ice‐cold RIPA lysis buffer (Solarbio, #R0010) supplemented with a protease inhibitor cocktail (Solarbio, #IKM1010) for 30 min on ice. The lysates were centrifuged at 12 000 × g for 15 min at 4°C, and the supernatants containing total protein were collected. Protein concentration was quantified with a BCA protein assay kit (Beyotime, #P00010). Aliquoted samples were snap‐frozen in liquid nitrogen and stored at −80°C until further analysis.

### 2.4 Cell Culture and Transfection

The SN4741 cell line, a murine dopaminergic neuronal model derived from the substantia nigra of the midbrain (RRID: CVCL_S466), was kindly provided by Dr. Pingyi Xu (Guangzhou Medical University, China). The cell line is not listed as a commonly misidentified cell line by the ICLAC (http://iclac.org/databases/cross‐contaminations/). Additionally, cell identity was authenticated by short tandem repeat profiling (conducted by Starfish BioTech, Suzhou, China), with the results confirming a match to the reference database. Cells were maintained at 37°C under 5% CO_2_ in high‐glucose Dulbecco's Modified Eagle Medium (DMEM; Gibco) supplemented with 10% heat‐inactivated fetal bovine serum (FBS; Gibco) and 1% penicillin‐streptomycin. Cells were routinely passaged when reaching 80%–90% confluence and were used within 30 passages. Treatments or transfections were typically performed when cells reached 50%–60% confluence. To establish an in vitro PD model, cells were exposed to MPP^+^ at concentrations of 0.5, 1.0, 1.5, or 2.0 mM. Following the manufacturer's instructions, cells were transfected with RNA oligonucleotides by using Lipofectamine 3000 reagent (Invitrogen, #L3000015). siRNA targeting mouse *HIF‐1*α (sequence: GGAGGACGAUGAACAUCAATT, Catalog number #HH20240622) was designed by Hanbio (China). Detailed siRNA sequences are provided in Supplementary Table 3. The mouse *HIF‐1*α overexpression plasmid and the *NDUFS1* promoter‐driven luciferase reporter plasmid were synthesized by Hanbio (China). SN4741 cells were plated in 6‐well plates and grown to 60%–70% confluence prior to transfection with the indicated constructs using Lipofectamine 3000 (Invitrogen, #L3000015). Total RNA and protein were harvested 48 h post‐transfection for RT‐qPCR and Western blot analyses, respectively.

### 2.5 Plasmid Construction and Transfection

The H4K12R mutant plasmid was constructed by GeneChem (Shanghai, China). Briefly, using wild‐type histone H4 cDNA as the template, site‐directed mutagenesis primers were used for PCR amplification. The amplified products were ligated into the vector, transformed into competent cells, and positive clones were screened and verified by Sanger sequencing. SN4741 cells were transfected with H4‐WT or H4‐K12R mutant plasmids using Lipofectamine 3000 (Invitrogen, #L3000015) following the manufacturer's instructions. Expression of the mutant protein was confirmed by Western blot analysis.

### 2.6 Establishment of the PD Mouse Model and Drug Treatment

A subacute PD mouse model was established using MPTP. Thirty male C57BL/6J mice (7‐8 weeks old, 22–25 g) were divided randomly into three groups: control group (phosphate‐buffered saline, PBS), MPTP group, and 2‐DG + MPTP group. Mice in the MPTP group received intraperitoneal injections of MPTP (30 mg/kg, Sigma–Aldrich, #M0896) once daily for 7 consecutive days. Mice in the 2‐DG + MPTP group were pretreated with 2‐DG (400 mg/kg, MCE, #HY‐13966) via intraperitoneal injection once daily for 3 days prior to MPTP administration. During the 7‐day MPTP injection period, 2‐DG was administered 2 h before each MPTP injection, resulting in a total of 10 days of 2‐DG treatment. Mice in the control group received an equivalent volume of PBS according to the same schedule. All mice were kept in a warm environment until fully recovered after each injection. To further validate the protective effect of PX‐478, we conducted an independent experiment using the MPTP‐induced PD model. Briefly, 28 male mice were randomly divided into four groups: control (PBS), MPTP, PX‐478, and PX‐478 + MPTP. Mice in the PX‐478 + MPTP group received intraperitoneal injections of PX‐478 (100 mg/kg) twice daily prior to MPTP administration and continued for 7 days post‐MPTP injection. Mice in the control and MPTP groups received an equivalent volume of PBS via intraperitoneal injection.

### 2.7 Behavioral Test

#### 2.7.1 Grasping Test

The grasping test was conducted to assess motor coordination and limb strength. A 1 mm diameter horizontal metal wire was securely fixed 30 cm above the ground. Each mouse was gently placed so that its forepaws grasped the wire, allowing the body to hang freely. The behavior was recorded for 10 s and scored according to the following criteria: falling within 10 s scored 0; gripping the wire with forepaws only scored 1; gripping with forepaws plus one hind limb scored 2; and gripping with both forepaws and both hind limbs scored 3. Each mouse was tested three times, and the average score from the three trials was used for statistical analysis.

#### 2.7.2 Pole Test

The pole test was performed to assess motor coordination and bradykinesia. A vertical wooden pole (50 cm in height, 1 cm in diameter) with a cork ball at the top was employed. Mice were trained for two consecutive days prior to testing, with three trials per day and an inter‐trial interval of at least 10 min. During the formal test, each mouse was placed head‐upward on the cork ball, and the time required to descend to the base of the pole was recorded. Each mouse performed three trials, and the mean latency was used for statistical analysis.

#### 2.7.3 Open‐Field Test (OFT)

Mice were placed and allowed to explore freely in an open‐field reaction box (30 cm in length, 30 cm in width, and 35 cm in height) for 5 min. The ANY‐MAZE video tracking system (Version 7.16, Stoelting, Wood Dale, IL) was used to record their movement distance and immobility time.

### 2.8 CUT&Tag

The CUT&Tag assay was conducted using the Hyperactive In‐Situ ChIP Library Prep Kit (pG‐Tn5, Vazyme Biotech, TD901) according to the manufacturer's protocol. Briefly, SN4741 cells were harvested and attached to Concanavalin A–coated magnetic beads. The cells were then suspended in antibody buffer and sequentially incubated with primary antibodies targeting H4K12la and corresponding secondary antibodies. Subsequently, pA‐Tn5 transposase was introduced to facilitate tagmentation. Following transposase activation, genomic DNA was extracted, PCR‐amplified, and purified for library preparation. Purification was performed using VAHTS DNA Clean Beads (N411, Vazyme Biotech). Library concentration was determined with the VAHTS Library Quantification Kit (Vazyme Biotech), and paired‐end sequencing (150 bp) was carried out on an Illumina NovaSeq platform.

### 2.9 Quantitative Chromatin Immunoprecipitation (qChIP)

Adherent SN4741 cells at the logarithmic growth phase were washed twice with ice‐cold PBS, crosslinked with 1% formaldehyde at room temperature for 10 min, and quenched with 125 mM glycine for 5 min. After two additional PBS washes, cells were resuspended in ice‐cold Buffer A, incubated on ice for 10 min, and centrifuged; pellets were then resuspended in Buffer B and digested with MNase (0–2000 gel units) at 37°C for 20 min before termination with EDTA. Chromatin was resuspended in ChIP Buffer, sonicated to obtain fragments of 200–500 bp, and treated sequentially with RNase A (37°C, 30 min) and Proteinase K (65°C, 2 h), with fragmentation efficiency verified by 1% agarose gel electrophoresis. Cleared lysates were diluted in ChIP Buffer (1% Triton X‐100, 2 mM EDTA, 150 mM NaCl, 20 mM Tris‐HCl, pH 8.1), pre‐cleared with Protein A/G magnetic beads (4°C, 30 min), and incubated overnight at 4°C with target‐specific antibodies or IgG control, followed by bead capture (4°C, 60 min). Beads were washed with low‐salt (150 mM NaCl), high‐salt (500 mM NaCl), LiCl (0.25 M), and TE buffers, then eluted twice with Elution Buffer (1% SDS, 0.1 M NaHCO3) at room temperature. Eluates and Input samples were reverse‐crosslinked with 200 mM NaCl at 65°C for 2 h, treated with 10 mM EDTA/40 mM Tris‐HCl/0.2 mg/mL Proteinase K (45°C, 1 h), and purified by phenol‐chloroform extraction followed by ethanol precipitation. DNA was resuspended in nuclease‐free water for qPCR analysis using Power SYBR Green Master Mix (Applied Biosystems) on a 7500 Real‐Time PCR System, with primer sequences provided in Supplementary Table 4.

### 2.10 Measurement of Lactate Levels

Frozen tissue samples from the substantia nigra and striatum of mice, or cultured SN4741 cells were homogenized in ice‐cold lysis buffer. The homogenates were sonicated on ice using a probe sonicator (300 W, 3 s pulse on/7 s pulse off for 3 min) and then centrifuged at 12 000 × g for 10 min at 4 °C. The supernatants were collected, and the lactate levels were determined using a Lactate Colorimetric Assay Kit (Biovision, #K607‐100) according to the manufacturer's protocol.

### 2.11 Quantitative Real‐Time RT‐PCR (RT‐qPCR)

Total RNA was extracted from the substantia nigra and striatum or SN4741 cells by TRIzol Reagent (Invitrogen) according to the manufacturer's instructions. Total RNA (1 µg) was used to synthesize cDNA with PrimeScript RT reagent Kit (Takara Bio, Japan). The resulting products were amplified on an Applied Biosystems 7500 Fast Real‐Time PCR System by SYBR Premix Ex Taq (Takara Bio, Japan). All results were normalized to GAPDH gene expression. The relative expression of targets was determined using the 2−ΔΔCT method. See Table S4 for primer sequences used for this analysis.

### 2.12 Seahorse Assay

Cellular oxygen consumption rate (OCR) was measured using a Seahorse XFe 24 Extracellular Flux Analyzer (Agilent Technologies). SN4741 cells were seeded in a Seahorse XF 24‐well microplate at a density of 5 × 10^4^cells per well and cultured overnight. Cells were then treated with 0.1 mM MPP^+^ for 24 h. Prior to the assay, cells were washed twice with XF assay medium. Mitochondrial respiratory function was assessed using the Seahorse XF Cell Mito Stress Test Kit (Agilent, #103015‐100) according to the manufacturer's protocol. Following baseline OCR measurements, mitochondrial modulators were injected sequentially to final concentrations as follows: 1 µM oligomycin (ATP synthase inhibitor), 2 µM carbonyl cyanide‐p‐trifluoromethoxyphenylhydrazone (FCCP; uncoupler), and a mixture of 1 µM rotenone and 1 µM antimycin A (Complex I and III inhibitors). OCR was recorded in real time and normalized to baseline values. Data were analyzed using Seahorse Wave Software and expressed as pmol O_2_ per minute.

### 2.13 Immunohistochemistry and Immunofluorescenc

Immunohistochemistry (IHC) was performed on paraffin‐embedded midbrain sections to detect TH‐positive dopaminergic neurons. Briefly, C57BL/6 and MPTP‐treated mice were transcardially perfused with PBS followed by 4% paraformaldehyde (PFA). Then brain tissues were post‐fixed, processed, and embedded in paraffin. Coronal sections (3 µm) containing the substantia nigra were cut and mounted on slides. These sections were then deparaffinized, rehydrated, and subjected to antigen retrieval in citrate buffer. To reduce background, endogenous peroxidase activity was blocked with 3% H_2_O_2_ for 30 min, and nonspecific binding was prevented by incubation with 5% normal goat serum containing 0.3% Triton X‐100 for 1 h. The sections were probed overnight at 4°C with an anti‐TH primary antibody, followed by a biotinylated secondary antibody and ABC complex. Staining was developed using DAB under visual monitoring, and nuclei were lightly counterstained with hematoxylin. Finally, sections were dehydrated, cleared, and cover slipped for brightfield imaging on an Olympus BX53 microscope. For immunofluorescence (IF) on tissue sections, paraffin‐embedded brain sections were similarly deparaffinized, rehydrated, and subjected to citrate‐based antigen retrieval. After blocking with 5% normal goat serum containing 0.3% Triton X‐100 for 30 min, sections were incubated with primary antibodies at 4 °C overnight, followed by fluorophore‐conjugated secondary antibodies at 37 °C for 1 h in the dark. Nuclei were stained with DAPI (7 min), and slides were mounted with anti‐fade medium. Images were acquired using a TCS SP8 laser‐scanning confocal microscope (Leica, Germany).

For cellular immunofluorescence, SN4741 cells were seeded on sterile coverslips in 12‐well plates. After senescence induction, cells were washed with PBS, fixed with 4% PFA for 10 min, and permeabilized with 0.1% Triton X‑100 for 10 min. Blocking was performed with 5% goat serum for 30 min, followed by incubation with primary antibodies overnight at 4 °C and fluorophore‐conjugated secondary antibodies for 1 h in the dark. Nuclei were counterstained with DAPI, and coverslips were mounted with anti‐fade medium. Confocal imaging was conducted on the same TCS SP8 system.

### 2.14 Metabolite Assays

Cells were harvested at a density of 1 × 10^6^ per sample, washed with ice‐cold PBS, and lysed according to the respective assay kit protocols. ATP levels were determined using the ATP Assay Kit (Beyotime, #S0027) following the chemiluminescence method. Luminescence was measured using a microplate luminometer, and ATP concentrations were calculated from a standard curve. For NAD^+^/NADH measurement, the NAD^+^/NADH Assay Kit (Colorimetric) (Beyotime, #S0176S) was utilized. Total NAD (NAD^+^ plus NADH) and NADH were measured separately; NAD^+^ content was calculated by subtracting NADH from total NAD levels. Briefly, cell extracts were prepared using the provided lysis buffer, and total NAD was measured colorimetrically at 450 nm following enzymatic conversion of NAD^+^ to NADH. For specific NADH quantification, samples were heated at 60°C for 30 min to deplete NAD^+^ prior to measurement. The NAD^+^/NADH ratio was calculated as (Total NAD − NADH)/NADH.

### 2.15 RNA‐seq Analysis

Total RNA was isolated from SN4741 cells following 24 h treatments with either MPP^+^ alone or MPP^+^ combined with *HIF‐1α* siRNA (three biological replicates per condition). All RNA sequencing services were conducted by Berry Genomics Corporation using established protocols. Quality‐filtered sequencing reads were mapped to the GRCh38 human genome assembly using TopHat (version 2.0.12) with default parameters. Gene‐level quantification was performed using HTSeq (v0.6.1) in union mode. Differential expression analysis was conducted with DESeq2 (v1.18.0) applying a Benjamini‐Hochberg adjusted p‐value threshold of 0.05 for significance. Functional annotation of differentially expressed genes was performed through Gene Ontology (GO) enrichment analysis using the GOseq algorithm (weighted method), with significantly enriched terms defined as those with FDR‐corrected p‐values <0.05. Pathway analysis was executed with KOBAS (v3.0) to identify statistically significant KEGG pathway associations (FDR<0.05).

### 2.16 Co‐Immunoprecipitation Assays (co‐IP)

Co‐IP was performed to assess the interaction between H4K12la and P300 using a commercial kit (Abcam, #ab206996). SN4741 cells were treated with MPP^+^ for 24 h, and cell lysates were prepared. According to the manufacturer's protocol, the lysates were incubated with specific antibodies overnight at 4 °C. Forty microliters of pre‐washed Protein A/G beads were then added to each sample and incubated at room temperature for 2 h on a rotator. After extensive washing, the immunoprecipitated complexes were eluted and analyzed by western blot.

### 2.17 ROS Measurement

Intracellular ROS levels in SN4741 cells were measured using fluorometric assays. The general ROS levels were assessed with the Reactive Oxygen Species Assay Kit (Beyotime, # S0033), while mitochondrial superoxide was specifically detected using MitoSOX Red Mitochondrial Superoxide Indicator (Thermo Fisher Scientific, # M36008).

For qualitative and semi‐quantitative analysis, cells were seeded in 12‐well plates. Following experimental treatments, cells were washed with PBS and incubated with the oxidation‐sensitive probe 2’,7’‐dichlorodihydrofluorescein diacetate (DCFH‐DA) or MitoSOX Red (5 µM) at 37°C in the dark for 20 or 10 min, respectively. As a positive control, a separate group of cells was pre‐treated with ROSup (2 µL/mL) for 15 min prior to DCFH‐DA staining. After incubation, cells were washed, and fluorescence images were acquired using a fluorescence microscope. The fluorescence intensity was quantified using ImageJ software (NIH, USA), with each condition analyzed in triplicate.

For quantitative analysis, SN4741 cells were seeded in 6‐well plates at a density of 0.8 × 10^6^ cells per well and cultured for 48 h. After treatment, cells were stained with 10 µM CM‐H_2_DCFDA (Invitrogen, #C6827) for 30 min at 37°C in the dark. Cells were then trypsinized, washed, and resuspended in FACS buffer (PBS containing 3% FBS). Flow cytometry was performed on a BD FACSCanto system (BD Biosciences, USA), collecting at least 10,000 events per sample. Data were analyzed using FlowJo software (BD Biosciences).

### 2.18 Mitochondrial Complex I Activity Assay

Mitochondrial complex I activity was determined using a Mitochondrial Complex I Activity Assay Kit (Solarbio, #BC0515) according to the manufacturer's protocol. Briefly, mitochondria were isolated from 5 × 10^6^ cells, and the enzymatic activity of complex I was quantified by monitoring NADH oxidation kinetics at 340 nm for 2 min using a spectrophotometer.

### 2.19 Western Blot

Protein extracts were prepared from brain tissues or cultured cells using RIPA lysis buffer supplemented with a complete protease and phosphatase inhibitor cocktail. Protein concentration was determined with a bicinchoninic acid (BCA) assay kit, and all samples were adjusted to equal concentrations prior to loading. Proteins were denatured at 95°C for 5 min, separated by sodium dodecyl sulfate–polyacrylamide gel electrophoresis (SDS‐PAGE), and subsequently transferred onto polyvinylidene difluoride (PVDF) membranes (Millipore). Membranes were blocked with 5% (w/v) non‐fat dry milk dissolved in Tris‐buffered saline containing 0.1% Tween‐20 (TBST) for 1 h at room temperature, then probed with specific primary antibodies at 4°C overnight. After three 10 min TBST washes, membranes were incubated with appropriate horseradish peroxidase (HRP)‐conjugated secondary antibodies for 1 h at room temperature. Protein bands were visualized using enhanced chemiluminescence (ECL) substrate and detected by autoradiography. Quantitative analysis was performed using ImageJ software (NIH), with band intensities normalized to respective loading controls. The primary antibodies used in this study were as follows: GAPDH Monoclonal antibody (60004‐1‐Ig; ProteinTech, China), Pan‐Kla (PTM‐1401RM; PTM Biolabs, China), H4K12la (PTM‐1411RM; PTM Biolabs, China), H3K18la (PTM‐1406RM; PTM Biolabs, China), H3K23la (PTM‐1413RM; PTM Biolabs, China), H3K9la (PTM‐1419RM; PTM Biolabs, China), H3K14la (PTM‐1414RM; PTM Biolabs, China), H3 (PTM‐1001RM; PTM Biolabs, China), TH (MAB318; Sigma‐Aldrich, USA), HIF‐1α (PA1‐16601; Thermo Fisher Scientific, USA), NDUFS1 (PTM‐20288; PTM Biolabs, China), ATPB (A5769; ABclonal Biotechnology, China), P300 (sc‐48343; Santa Cruz Biotechnology, USA), β‐actin (4970; Cell Signaling Technology, USA), IBA1 (RT1316; BUABIO, China), HK2 (66974‐1‐Ig; ProteinTech, China), LDHA (R24822; Zenbio, China).

### 2.20 Molecular Docking

The molecular docking was used to predict the potential binding between H4K12la and P300. The structures of H4K12la and P300 were obtained from PubChem (CID: 31553) and Protein Data Bank (PDB ID: 3AJO), respectively. Discovery studio software (BIOVIA, San Diego, USA) was utilized for binding site analysis and docking. The binding affinity of H4K12la with P300 was evaluated using the LibDock score LibDock score.

### 2.21 Dual Luciferase Report Gene Detection

The predicted HIF‐1α binding sequences within the *NDUFS1* promoter region, along with their corresponding mutant variants, were chemically synthesized and subsequently cloned into pGL3‐basic luciferase reporter vectors. Luciferase activity was determined using the Dual‐Lumi Luciferase Assay System (Beyotime, RG089S) in strict accordance with the manufacturer's recommended procedures. Relative luminescence units were quantified employing a Synergy 2 multi‐mode microplate reader (BioTek Instruments, USA). All experimental conditions were independently replicated a minimum of three times. The complete nucleotide sequences of both wild‐type and mutant constructs have been compiled in Supplementary Table 5.

### 2.22 Statistical Analyses

Western blot and immunofluorescence results were quantitatively analyzed for grayscale intensity using ImageJ software (National Institutes of Health, USA). Flow cytometry data were processed and statistically analyzed using FlowJo software (FlowJo LLC, USA). All acquired continuous data were tested for normality. Data are expressed as the mean ± standard deviation (mean ± SD) or the mean ± standard error of the mean (mean ± SEM), while categorical data from clinical samples are presented as numbers and percentages. Statistical analysis and graphing were performed using IBM SPSS Statistics version 24.0 (IBM Corp, USA) and GraphPad Prism version 9.0 (GraphPad Software, USA). For normally distributed continuous data, comparisons between two independent groups were made using unpaired two‐tailed Student's t‐tests, while comparisons among multiple groups were conducted using one‐way analysis of variance (ANOVA). For categorical variables, group comparisons were performed using the Chi‐square test or Fisher's exact test, as appropriate. Fisher's exact test was used when the expected frequency in any cell of the contingency table was less than 5. A p‐value of less than 0.05 was considered statistically significant.

## 3 Results

### 3.1 Lactate Levels and Histone Lactylation Are Elevated in PD Patients

To investigate lactate metabolism in PD, we first compared resting plasma lactate levels between 83 PD patients and 83 HCs with well‐matched baseline characteristics (Table S1). Significantly higher resting lactate levels were observed in PD patients than in HCs (Figure 1A). Given the influence of physical exertion on lactate, a subgroup (n = 31 per group) underwent a simplified lactate exercise test. Although post‐exercise lactate levels were higher in the HC group (Figure 1B), the difference between the two groups was reduced after 10 min of rest (Figure 1C). Furthermore, the change in lactate levels from post‐exercise to post‐rest was significantly greater in HCs than in PD patients (Figure 1D), collectively indicating impaired lactate clearance in PD. We next examined the clinical relevance of resting lactate within the PD cohort. Resting lactate levels exhibited a significant positive correlation with motor severity, as assessed using UPDRS‐III scores (Figure 1E). Consistent with this finding, patients with moderate and advanced H&Y stages (3–5) had significantly higher lactate levels than those with early stages (1–2.5) (Figure 1F), and there was a positive correlation between lactate levels and H&Y stage (Figure 1G). By contrast, no significant correlations were identified between lactate levels and disease duration or levodopa equivalent dose (Figure 1H,I).

**FIGURE 1:**
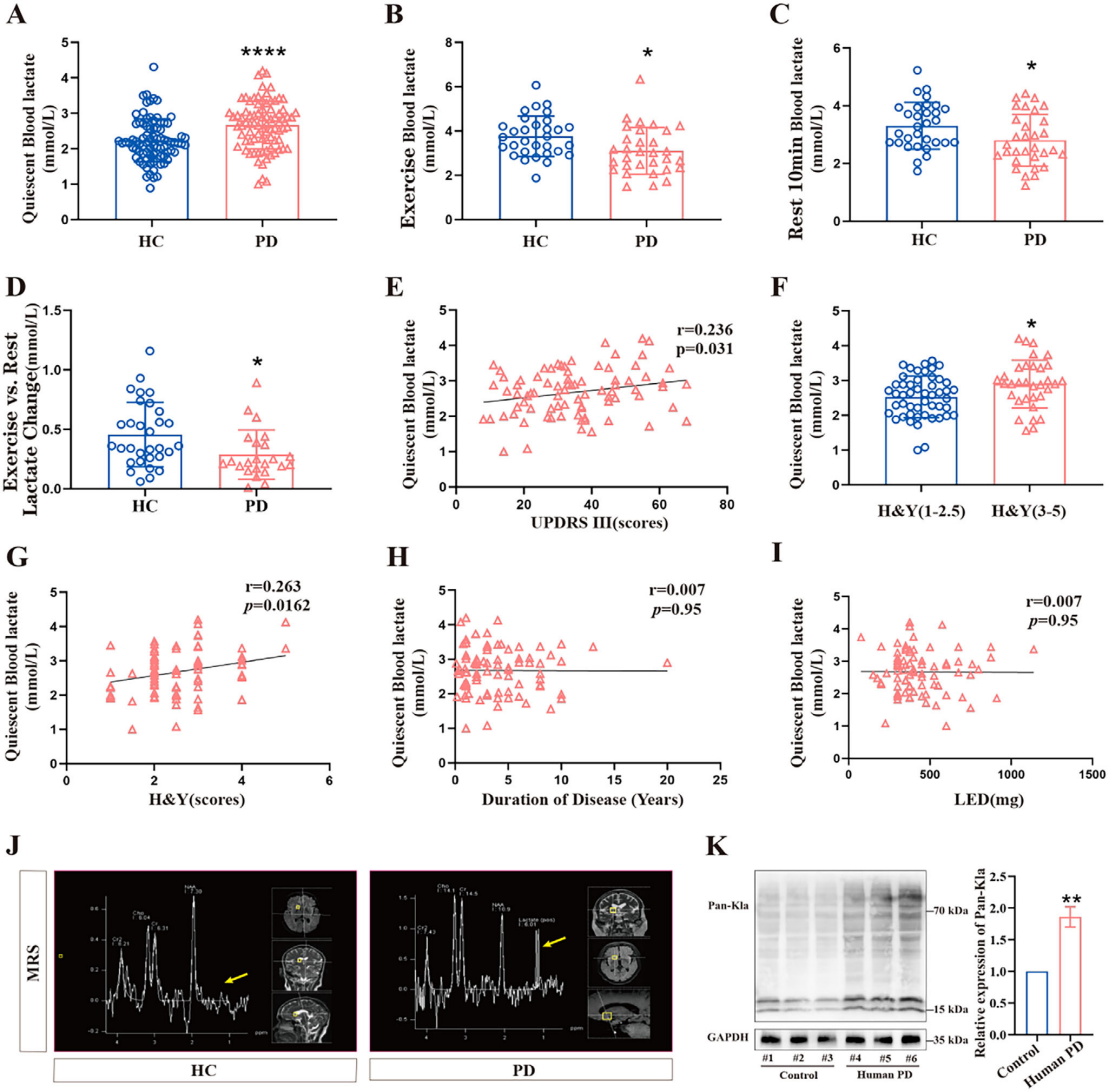
Systemic Lactate Metabolic Dysregulation in Parkinson's Disease (PD). (A) Resting plasma lactate levels in PD patients (n = 83) and healthy controls (HCs, n = 83). (B,C) Lactate levels in a subgroup (n = 31). (B) immediately after a simplified exercise test and (C) 10 min after rest. (D) The change in lactate levels (Δlactate) from post‐exercise to 10 min after rest. (E) Correlation between resting lactate levels and UPDRS‐III motor scores in the PD group (n = 83). (F) Resting lactate levels in PD patients stratified by early (H&Y 1–2.5) and advanced (H&Y 3–5) disease stages. (G) Correlation between resting lactate levels and H&Y stage in the PD group (n = 83). (H,I) Correlation between resting lactate levels and (H) disease duration or (I) LED in the PD group. (J) Proportion of subjects with a detectable lactate peak in the striatum as measured by magnetic resonance spectroscopy (MRS) (PD, n = 36; HCs, n = 13). (K) Western blot analysis of global pan‐lysine lactylation levels in PBMCs from PD patients and HCs (n = 6). ^*^ *p* < 0.05, ^**^ *p* < 0.01, ^***^ *p* < 0.001, ^****^ *p* < 0.0001. Data are presented as mean ± SEM (A–D,F,K). Statistical significance was determined by two‐tailed unpaired Student's t‐test (A–D,F,K), Spearman correlation analysis (E,G–I), and Fisher's exact test (J). UPDRS, unified Parkinson's disease rating scale; LED, Levodopa Equivalent Dose.“n” refers to the number of participants.

Next, we used MRS to assess cerebral lactate. A detectable lactate peak in the striatum was observed in a significantly higher proportion of PD patients than of HCs (Figure 1J and Table S2). We then evaluated global protein lactylation, with a focus on histone lactylation, in peripheral blood mononuclear cells (PBMCs). Histone lactylation levels were significantly higher in PD patients than in HCs (Figure 1K). These results collectively indicate that aberrant lactate metabolism is an important feature of PD that is linked to motor severity and manifested through impaired clearance, central accumulation, and elevated histone lactylation.

### 3.2 H4K12la Levels Are Specifically Elevated in PD Models

To investigate the role of lactate and histone lactylation in PD pathogenesis, we first established an MPTP‑induced PD mouse model [26]. Successful modeling was confirmed by a significant reduction in TH expression in striatum and substantia nigra (Figure 2A–C). Compared with controls, PD mice exhibited markedly elevated lactate levels in both the striatum and substantia nigra (Figure 2D). To determine whether this lactate originates from enhanced central glycolysis rather than peripheral sources, we examined the expression of key glycolytic enzymes in the substantia nigra of MPTP‐induced mice. Western blot analysis revealed that both hexokinase 2 (HK2) and lactate dehydrogenase A (LDHA) were significantly upregulated in the substantia nigra and striatum of MPTP‑treated mice compared with controls (Figure S1A–D). To determine whether these upregulated glycolytic enzymes are enriched within dopaminergic neurons, we performed immunofluorescence co‑staining for HK2/LDHA and TH in the substantia nigra. The results showed that both HK2 and LDHA were markedly increased specifically within residual TH^+^ dopaminergic neurons (Figure S1E–H). Given that lactate overproduction provides the crucial substrate for histone lactylation [8], we then assessed lactate and protein lactylation levels within our PD mouse model. Consistent with the increase in lactate, western blot analysis revealed an increase in global protein lactylation, especially in histone lactylation (Figure 2E,F).

**FIGURE 2:**
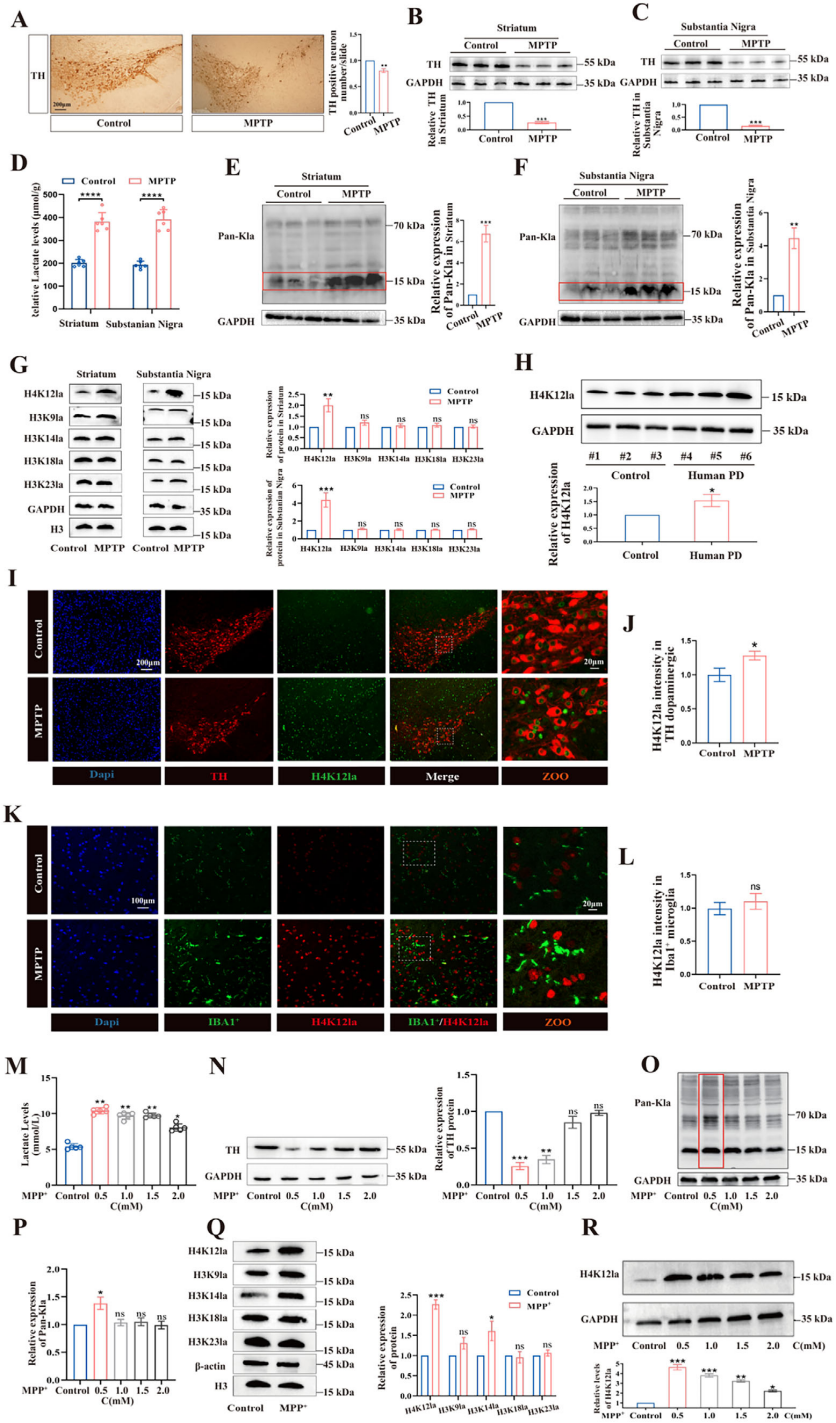
Characterization of lactate levels and histone lactylation in MPP^+^‐treated cellular and MPTP‐induced mouse models of PD. (A) Representative immunohistochemical images and quantitative analysis of TH expression in the midbrain of MPTP‐induced PD mice and controls (n = 3). Scale bar, 200 µm. (B,C) Western blot analysis and quantification of TH protein levels in the (B) striatum and (C) substantia nigra of MPTP‐induced PD mice (n = 3). (D) Lactate levels in the striatum and substantia nigra homogenates of MPTP‐induced PD mice compared to controls (n = 3). (E,F) Western blot analysis of Pan‐Kla levels in both the (E) striatum and (F) substantia nigra of MPTP‐induced PD mice compared to controls (n = 3). (G) Western blot analysis and quantification of H4K12la, H3K9la, H3K14la, H3K18la, H3K23la protein levels in both the striatum and substantia nigra of MPTP‐induced PD mice compared to controls (n = 3). (H) Representative Western blot analysis of H4K12la from cryopreserved PBMCs of 18 PD patients and 18 HCs. (I,J) Representative immunofluorescence images of midbrain sections showing a marked decrease in TH signal (red) and a concurrent increase in Pan‐Kla signal (green) in MPTP‐treated mice. Scale bars: left, 200 µm; right, 20 µm. (n = 3). (K,L) Representative immunofluorescence images of midbrain sections showing a marked decrease in IBA1^+^ signal (green) and a concurrent elevation of H4K12la signal (red) in MPTP‐treated mice. Scale bars: left, 100 µm; right, 20 µm. (n = 3). (M) Changes in extracellular lactate levels under treatment with different concentrations of MPP^+^ (0.5, 1.0, 1.5, and 2.0 mM) (N = 3). (N) Changes in the expression levels of TH protein under treatment with different concentrations of MPP^+^ (0.5, 1.0, 1.5, and 2.0 mM) (N = 3). (O,P) Western blot analysis and quantification of Pan‐Kla protein levels under treatment with different concentrations of MPP^+^ (0.5, 1.0, 1.5, and 2.0 mM) (N = 3). (Q) The expression levels of multiple histone lactylation sites (H4K12la, H3K18la, H3K9la, H3K14la, and H3K23la) in SN4741 cells treated with or without 0.5 mM MPP^+^ were detected by Western blotting (N = 3). (R) The expression level of H4K12la in SN4741 cells treated with different concentrations of MPP^+^ (0.5, 1.0, 1.5, and 2.0 mM) was detected by Western blotting (N = 3). Data are mean ± SEM. ns, not significant, ^*^ *p* < 0.05, ^**^ *p* < 0.01, ^***^ *p* < 0.001, and ^****^ *p* < 0.0001. Statistical significance was determined by one‐way ANOVA or Student's t‐test as appropriate. TH, Tyrosine Hydroxylase; IBA1+, Ionized Calcium‐Binding Adapter Molecule 1; “n” refers to the number of mice per group; “N” refers to the number of biologically independent experiments.

Because histone lactylation has broad regulatory roles [12, 15, 27], we next sought to identify the altered lactylation site(s) in the PD model. The screening of several candidate sites (including H4K12la, H3K18la, H3K9la, H3K14la, and H3K23la) revealed that H4K12la was the most prominently increased histone lactylation mark in the striatum and substantia nigra of MPTP‐induced mice (Figure 2G). Separately, we examined H4K12la levels in peripheral blood samples from PD patients and healthy controls. Western blot analysis confirmed that H4K12la levels were significantly higher in the PD group than in the controls (Figure 2H). To examine the cellular localization of the increased H4K12la, we performed immunofluorescence co‐staining for H4K12la in combination with specific markers: TH for dopaminergic neurons and IBA1 for microglia. Colocalization analysis revealed a marked increase in H4K12la levels in TH‐positive dopaminergic neurons, whereas no significant alterations were detected in microglia (Figure 2I–L). Collectively, these in vivo data indicate that MPTP exposure induces a systemic increase in lactate levels and histone lactylation (notably H4K12la) in dopaminergic brain regions and suggest that this modification occurs predominantly in neurons rather than in microglia.

To validate and extend the in vivo findings, we used the dopaminergic neuronal cell line SN4741 to establish an MPP^+^‑induced PD cell model. First, we evaluated the effects of different concentrations of MPP^+^ (0.2, 0.4, 0.8, 1.0, 2.0, and 3 mM) on SN4741 cell viability using a CCK‐8 assay kit. We then plotted a standard curve, from which the half‐maximal inhibitory concentration (IC_50_) of MPP^+^ was determined as 1.1 mM (Figure S1I). We then selected four concentration gradients around the IC_50_ value (0.5, 1.0, 1.5, and 2.0 mM) to optimize the model construction conditions. Of these, 0.5 mM MPP^+^ most effectively increased extracellular lactate levels (Figure 2M) and decreased TH expression (Figure 2N). Consistent with the lactate overproduction data, western blot analysis demonstrated a rise in global protein lactylation—especially histone lactylation—in the MPP^+^‐induced cells (Figure 2O,P). We further confirmed that H4K12la was the most prominently upregulated histone lactylation site in the MPP^+^‐induced SN4741 cells (Figure 2Q). Additionally, dose–response experiments across MPP^+^ concentrations (0.5–2.0 mM) revealed that 0.5 mM MPP^+^ was the most effective condition for upregulating H4K12la (Figure 2R), thereby validating the stability and specificity of this modification in the cell model. Collectively, these data indicate that H4K12la levels are specifically elevated in both in vitro and in vivo PD models, and are accompanied by dopaminergic neuron loss. These findings suggest a potential role for H4K12la in PD pathogenesis; further investigations into its underlying mechanisms and therapeutic implications are therefore warranted.

### 3.3 Genome‐Wide Analysis of the Transcriptional Consequences of H4K12la in SN4741

Next, we sought to explore the functional importance of H4K12la in dopaminergic neurons in PD. As a newly identified epigenetic modification, histone lactylation has been reported to directly regulate gene transcription at the chromatin level. We therefore performed a genome‐wide CUT&Tag analysis using an anti‐H4K12la antibody; data were analyzed using deepTools (Figure 3A) [12]. In the MPP^+^‐induced group, there was a noticeable enrichment of H4K12la peaks (Figure 3B,C). The dopaminergic neuron comparison revealed 13 194 differential H4K12la binding peaks, of which 29.43% were located within promoter sequences (≤3 kb) (Figure 3D). We then analyzed the functional enrichment of 4321 genes that were targeted by promoter‐associated H4K12la in PD models. These genes were significantly enriched in metabolic processes (GO analysis, Figure 3E) and in KEGG pathways such as HIF‐1α signaling and oxidative phosphorylation–related metabolism (Figure 3F).

**FIGURE 3:**
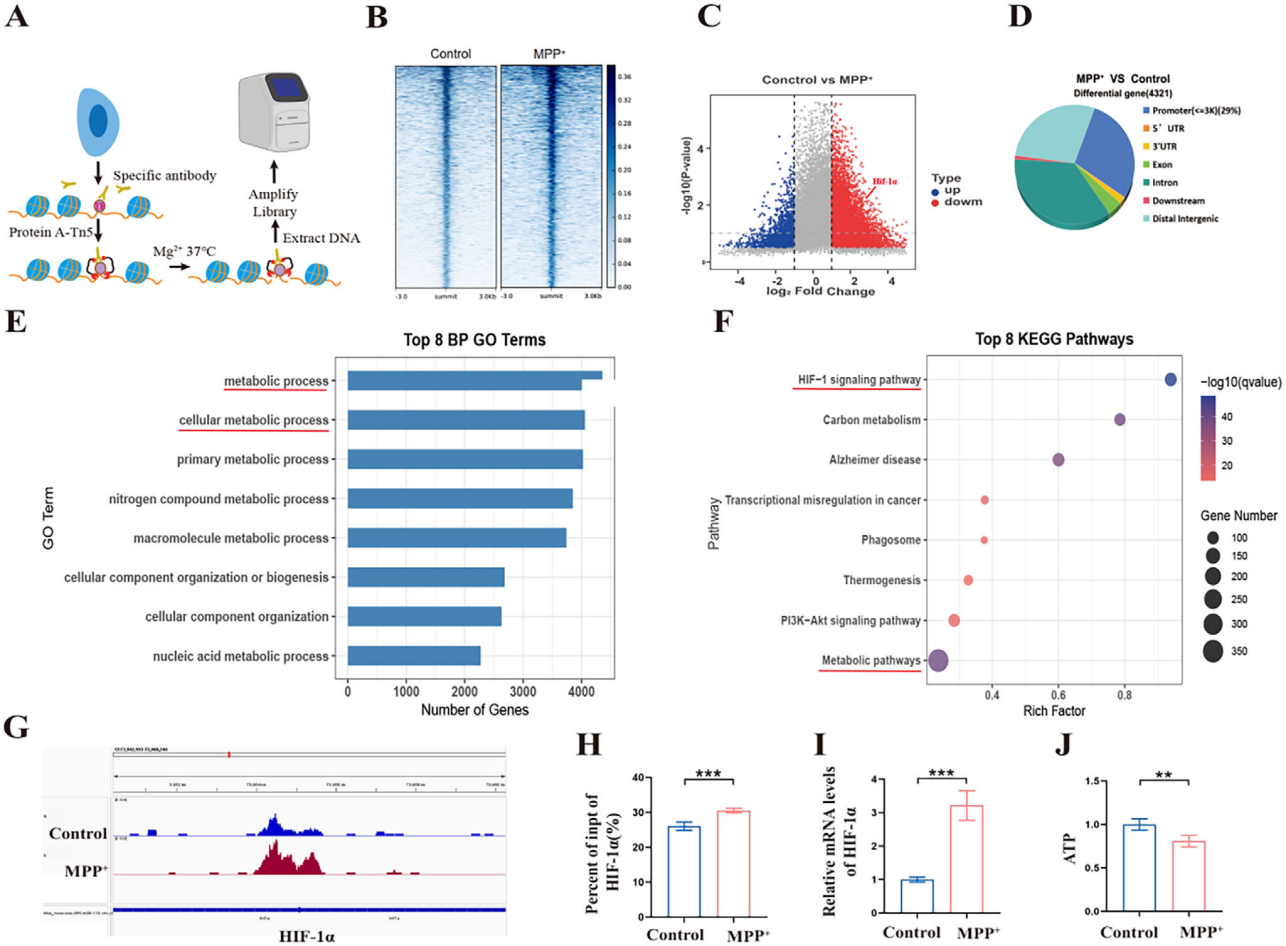
Genome‐wide profiling of H4K12la reveals its role in regulating metabolic genes in a cellular PD model. (A) Schematic workflow of the CUT&Tag assay for H4K12la in SN4741 cells treated with or without MPP^+^. (B) The binding density of H4K12la was visualized by deepTools. The heatmap presents the CUT&Tag tag counts on H4K12la binding peaks in control and MPP^+^‐treated SN4741 cells, ordered by signal strength. (C) Volcano plots illustrate differentially expressed genes in SN4741 cells treated with or without MPP^+^. (D) The distribution of H4K12la on the genome. (E) GO enrichment analysis of biological processes for genes associated with promoter‐bound H4K12la peaks in MPP^+^‐treated SN4741 cells. (F) KEGG pathway enrichment analysis for genes associated with promoter‐bound H4K12la peaks. (G) Genome browser tracks displaying CUT&Tag signals at the representative *HIF‐1α* gene locus. (H) qChIP analysis of the *HIF‐1α* promoter was performed using antibodies against H4K12la from Control and MPP^+^‐treated SN4741 cells (N = 3). (I) RT‐qPCR analysis of *HIF‐1α* mRNA expression levels from Control and MPP^+^‐treated SN4741 cells (N = 3). (J) ATP levels inside the cell from Control and MPP^+^‐treated (N = 3). Data are presented as mean ± SEM. ^**^ *p* < 0.01, and ^***^ *p* < 0.001. Two‐tailed unpaired Student's t test (H–J). GO, Gene Ontology; KEGG, Kyoto Encyclopedia of Genes and Genomes; RT‐qPCR, reverse transcription quantitative PCR, qChIP, quantitative chromatin immunoprecipitation; “N” refers to the number of biologically independent experiments.

Given the central role of the HIF‐1α pathway in metabolic reprogramming and its significant enrichment among H4K12la‐targeted genes, we hypothesized that HIF‐1α itself might be a direct transcriptional target of H4K12la. Consistent with this hypothesis, H4K12la levels were specifically increased at the promoters of associated genes, including HIF‐1α (Figure 3G), thus underscoring its role in these regulatory networks. Quantitative chromatin immunoprecipitation analysis revealed significantly elevated H4K12la levels at *HIF‐1α* promoters (Figure 3H). Consistent with this finding, RT‐qPCR demonstrated elevated *HIF‐1α* mRNA levels in MPP^+^‐induced SN4741 cells (Figure 3I). Because HIF‐1α is a master regulator of glycolytic metabolism, we next assessed the functional consequences of its upregulation on cellular energy homeostasis. We observed a concomitant decrease in intracellular ATP content (Figure 3J), indicating an associated disruption of energy metabolism. Collectively, these results suggest that H4K12la modification activates HIF‐1α transcription, thereby contributing to metabolic dysfunction in PD cell models.

### 3.4 H4K12la Regulates HIF‐1α Expression in PD Models

Because the gene sequence that controls histone 4 editing is highly conserved, it is difficult to modulate it using a CRISPR/Cas9‐based technique [28]. We therefore provided a lactyl group by co‐culture in cell media to replace the over‐lactylation of the protein [29]. To investigate whether HIF‐1α expression is regulated by H4K12la, we treated SN4741 cells separately with MPP^+^ (0.5 mM), lactate, or sodium lactate (NaLa). Compared with controls (NaCl or untreated), HIF‐1α protein expression levels were significantly increased in the MPP^+^, lactate, and NaLa groups, and exhibited a consistent trend with H4K12la expression levels (Figure 4A). Furthermore, immunofluorescence analysis revealed that MPP^+^, lactate, and NaLa treatments enhanced the nuclear signals of both H4K12la and HIF‐1α in SN4741 cells (Figure 4B,C). At the transcriptional level, RT‐qPCR results demonstrated a parallel upregulation of *HIF‐1*α mRNA in the three treatment groups (Figure S2A).

**FIGURE 4:**
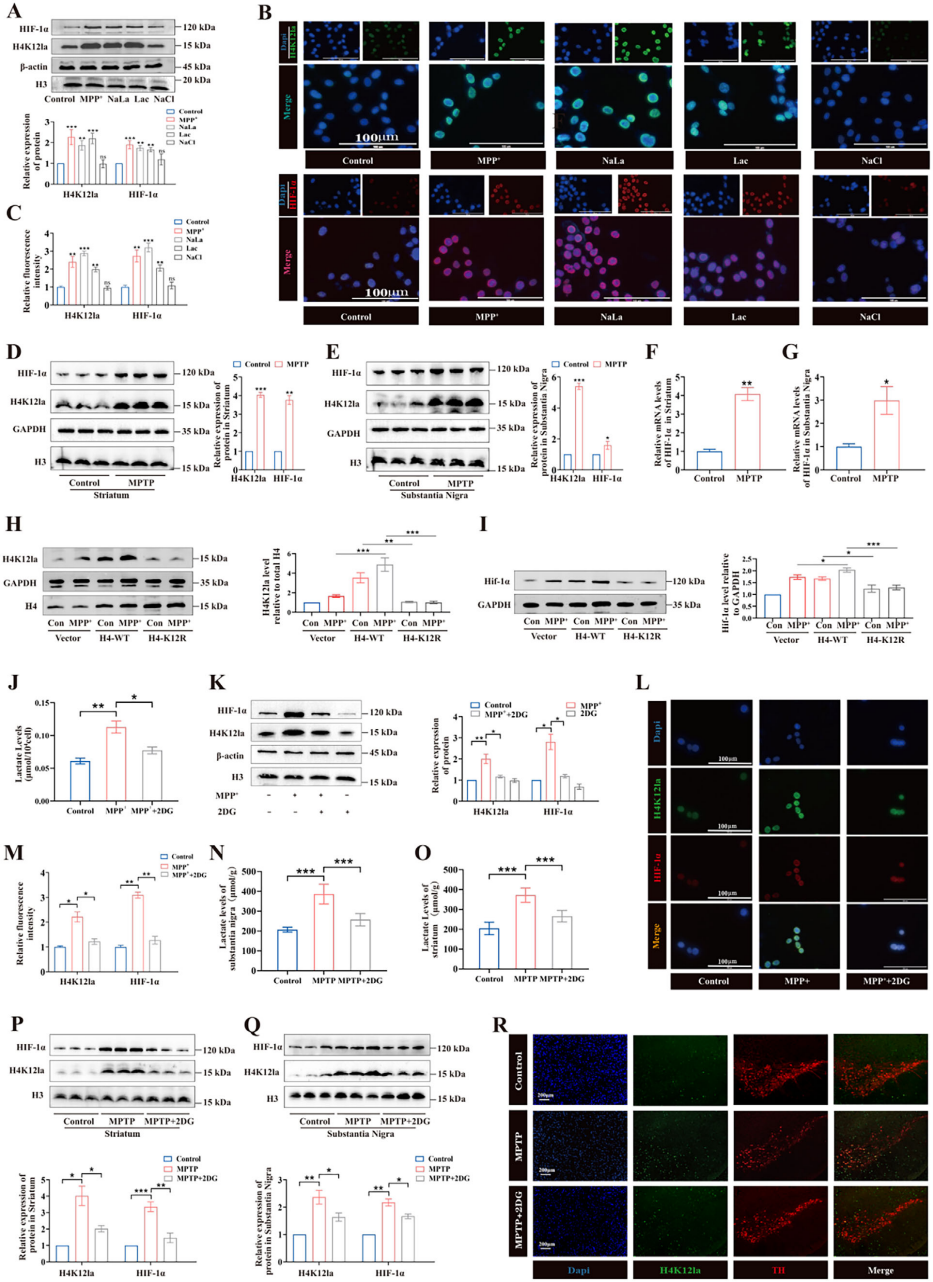
H4K12la regulates HIF‐1α expression in PD models, and inhibition of lactylation by 2‐DG ameliorates related pathologies. (A) Western blotting analysis of H4K12la and HIF‐1α protein levels in SN4741 cells treated with 0.5 mM MPP^+^, NaLa, Lac, or NaCl for 24 h (N = 3). (B) Representative immunofluorescence images of SN4741 cells showing H4K12la (green) and HIF‐1α (red) expression in the nucleus under the indicated treatments. DAPI (blue) stains nuclei. Scale bar, 100 µm. Insets show 2 × magnifications of the boxed regions. (C) Quantitative analysis of the relative fluorescence intensity of H4K12la and HIF‐1α. (D,E) Western blotting analysis and quantification of H4K12la and HIF‐1α protein levels in the (D) striatum and (E) substantia nigra of MPTP‐induced PD mice (n = 3). (F, G) RT‐qPCR analysis of *HIF‐1α* mRNA levels in the (F) striatum and (G) substantia nigra of MPTP‐induced PD mice (n = 3). (H,I) Cells transfected with H4‐WT or H4‐K12R were treated with MPP^+^. H4K12la and HIF‐1α protein levels were assessed by Western blotting (N = 3). (J) Intracellular lactate levels in MPP^+^‐treated SN4741 cells with or without 2‐DG treatment (N = 3). (K) Western blotting analysis of H4K12la and HIF‐1α protein levels in MPP^+^‐treated SN4741 cells with or without 2‐DG co‐treatment (N = 3). (L) Representative immunofluorescence images of SN4741 cells showing that 2‐DG co‐treatment reduces MPP^+^‐treated elevations of H4K12la (green) and HIF‐1α (red). Scale bar, 100 µm. (M) Corresponding quantification of fluorescence intensity for H4K12la and HIF‐1α. (N,O) Lactate levels in the (N) substantia nigra and (O) striatum homogenates of MPTP‐induced PD mice with or without 2‐DG treatment (n = 3). (P,Q) Western blotting analysis and quantification of H4K12la and HIF‐1α protein levels in the (P) striatum and (Q) substantia nigra of MPTP‐induced PD mice with or without 2‐DG treatment (n = 3). (R) Representative immunofluorescence images of midbrain sections from MPTP‐induced PD mice with or without 2‐DG treatment, showing TH (red) and H4K12la (green) expression. Scale bar, 200 µm (n = 3). Data are presented as mean ± SEM. ns, not significant, ^*^ *p* < 0.05, ^**^ *p* < 0.01, and ^***^ *p* < 0.001. Statistical significance was determined by one‐way ANOVA or Student's t‐test as appropriate. Lac, lactate; NaLa, sodium lactate; NaCl, sodium chloride; 2‐DG, 2‐deoxy‐D‐glucose. “n” refers to the number of mice per group. “N” refers to the number of biologically independent experiments.

Building on our in vitro findings, we next investigated the expression levels of HIF‐1α and H4K12la in vivo. A significant elevation in HIF‐1α levels was observed in both the substantia nigra and corpus striatum of MPTP‐treated mice compared with control mice, and this was accompanied by an upregulation of H4K12la (Figure 4D,E). This increase was also consistent at the transcriptional level, as evidenced by elevated *HIF‐1α* mRNA (Figure 4F,G). Together, these data suggest that elevated lactylation levels of H4K12 can promote HIF‐1α expression in a PD model.

To further establish a causal link between H4K12la and HIF‐1α, we adopted site‐directed mutagenesis approach by generating plasmids expressing wild‐type H4 (H4‐WT) and the H4K12‐to‐arginine substitution mutant (H4‐K12R), and transfected them into SN4741 cells. The constructs were verified by Sanger sequencing analysis (Figure S2B). As expected, the H4K12R mutation effectively abrogated H4K12la modification under both basal and lactate‐stimulated conditions (Figure S2C), confirming the loss of lactylation at this site. Moreover, H4‐WT cells exhibited elevated HIF‐1α protein and mRNA levels compared with H4‐K12R cells under both basal and lactate‐treated conditions, and the lactate‐induced upregulation of HIF‐1α was abolished in the mutant cells (Figure S2D,E).

We next asked whether this mechanism also contributes to MPP^+^‐induced HIF‐1α accumulation under pathological metabolic disturbance. As shown in Figure 4H,I and Figure S2F, expression of the H4‐K12R mutant significantly reduced H4K12la modification levels and suppressed MPP^+^‐induced upregulation of HIF‐1α protein and mRNA compared with H4‐WT cells. These results, together with the lactate stimulation data, support a causal role of H4K12la in HIF‐1α transcriptional activation.

To further corroborate this causal relationship, we also inhibited lactate production using the glycolysis inhibitor 2‐deoxy‐D‐glucose (2‐DG) [30]. In MPP^+^‐treated SN4741 cells, 2‐DG effectively decreased intracellular lactate (Figure 4J). To determine whether 2‑DG selectively reduces H4K12la or broadly suppresses multiple histone lactylation sites, we examined the levels of several candidate lactylation marks in MPP^+^‑treated SN4741 cells following 2‑DG treatment. Western blot analysis showed that 2‑DG significantly reduced H4K12la (Figure 4K), but did not alter the levels of H3K18la, H3K9la, H3K14la, or H3K23la (Figure S2G). These results indicate that, under our experimental conditions, 2‑DG selectively reduces H4K12la without broadly affecting other histone lactylation sites. Consistent with the reduction in H4K12la, 2‑DG also decreased HIF‑1α protein (Figure 4L,M) and *HIF‑1α* mRNA (Figure S2H). In vivo, 2‐DG administration to MPTP‐induced mice significantly reduced lactate levels in striatal and nigral tissue (Figure 4N,O). Moreover, western blot analyses demonstrated that 2‐DG treatment reversed the MPTP‐induced elevations of both H4K12la and HIF‐1α in mouse brain tissue (Figure 4P,Q). Consistent with the western blot data, immunofluorescence analysis confirmed a reduction in H4K12la signals in the midbrain with 2‐DG treatment (Figure 4R and Figure S2I). To further validate the upstream relationship of H4K12la to HIF‑1α in vivo, we performed co‑immunofluorescence staining for H4K12la and HIF‑1α in the substantia nigra of control, MPTP‑treated, and MPTP+2‑DG‑treated mice. In MPTP mice, both signals were elevated and colocalized in dopaminergic neurons, and 2‑DG treatment reduced both modifications (Figure S2J,K). These in vivo data support that H4K12la acts upstream of HIF‑1α, consistent with our cellular findings.

Collectively, by both increasing lactate availability (via exogenous lactate/NaLa) and inhibiting endogenous lactate production (via 2‐DG), together with the genetic evidence from site‐specific mutagenesis, we demonstrate that H4K12la positively regulates HIF‑1α expression in PD models.

### 3.5 Inhibition of H4K12la Rescues Metabolic Dysfunction and Dopaminergic Neuron Loss in PD Models

Because our findings indicated that H4K12la upregulates HIF‐1α, we next assessed the functional consequences on cellular energy metabolism. NAD^+^ serves as a critical electron carrier in oxidative phosphorylation; it facilitates the transfer of reducing equivalents (H^+^ and e^−^) to the ETC to drive ATP synthesis. The simultaneous measurement of the NAD^+^/NADH ratio and ATP levels therefore provides a robust assessment of cellular energy metabolism [31].

MPP^+^ treatment significantly reduced both ATP levels and the NAD^+^/NADH ratio in SN4741 cells. Notably, 2‐DG co‐treatment reversed these metabolic alterations (Figure 5A,B). To explore the effects of 2‐DG on energy metabolism and behavioral performance in mice, we intraperitoneally injected 2‐DG followed by MPTP administration, and subsequently conducted behavioral tests prior to sacrifice (Figure 5C). 2‐DG treatment significantly reversed the MPTP‐induced decline in both the NAD^+^/NADH ratio and ATP levels in the substantia nigra and striatum of mice (Figure 5D–G). Next, we assessed whether this molecular intervention translates to neuroprotection. Strikingly, 2‐DG treatment significantly mitigated the MPTP‐induced loss of dopaminergic neurons, as evidenced by a significant rescue of TH^+^ neurons in the midbrain (Figure 5H). Mechanistically, this recovery was associated with the restoration of mitochondrial ATP production capacity, which was indicated by the reversal of the MPTP‐induced downregulation of ATP synthase subunit beta in the substantia nigra and striatum (Figure 5I,J). Accordingly, *TH* mRNA expression in the substantia nigra, which was downregulated by MPTP, was also restored by 2‐DG treatment (Figure 5K).

**FIGURE 5:**
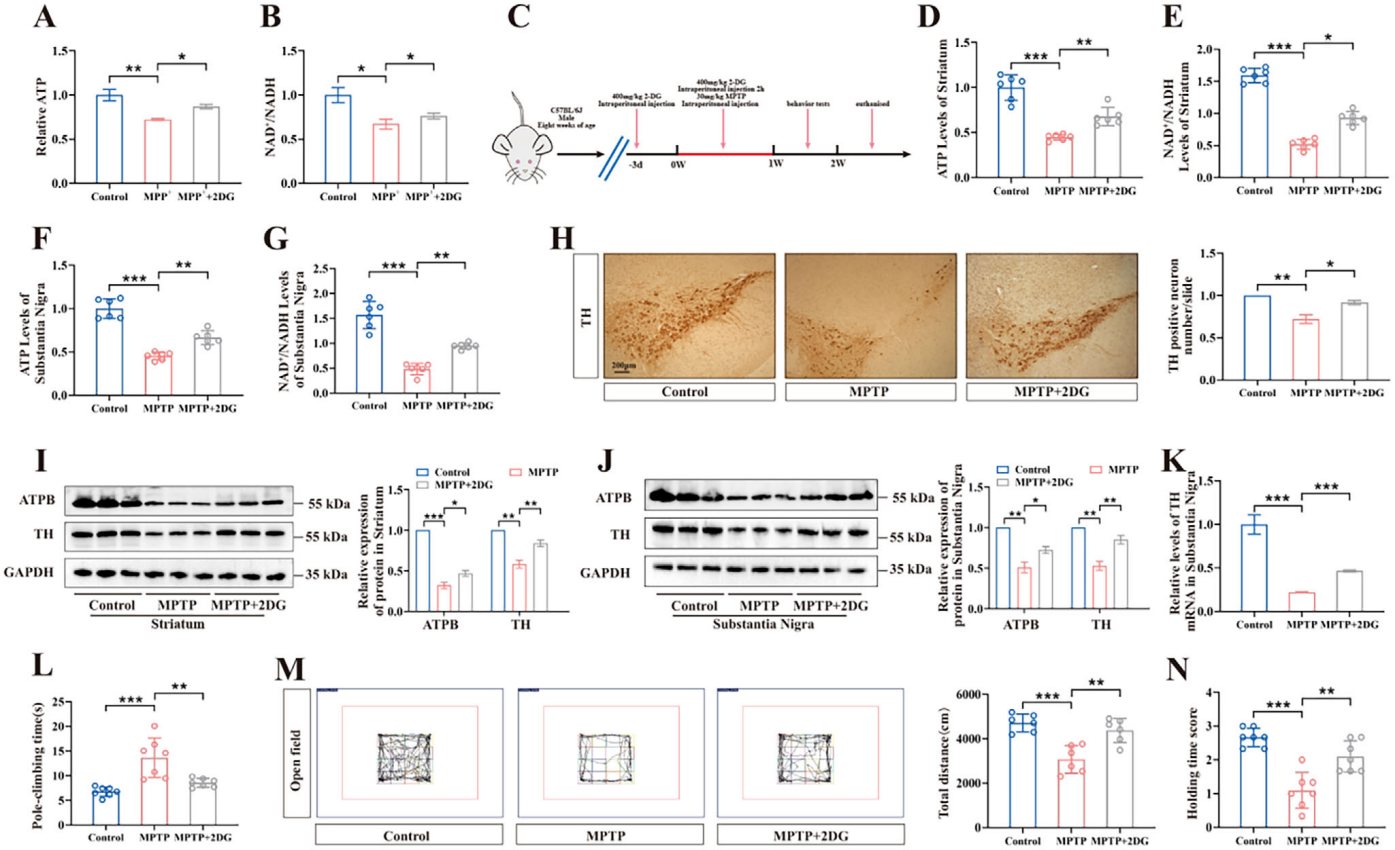
2‐DG ameliorates energy metabolism deficits, provides neuroprotection, and improves motor function in PD models by regulating H4K12la. (A,B) Measurement of (A) the ATP levels and (B) NAD^+^/NADH ratio in SN4741 cells treated with MPP^+^, with or without 2‐DG (N = 3). (C) Experimental schedule. (D–G) Measurement of the ATP levels and NAD^+^/NADH ratio in the (D,E) striatum and (F,G) substantia nigra of MPTP‐induced mice treated with or without 2‐DG (n = 3). (H) Representative immunohistochemical images and quantitative analysis of TH expression in the midbrain of mice treated with MPTP, with or without 2‐DG (n = 3). Scale bar, 200 µm. (I,J) Western blotting analysis and quantification of ATPB protein levels in the (I) striatum and (J) substantia nigra of mice treated with MPTP, with or without 2‐DG (n = 3). (K) RT‐qPCR analysis of *TH* mRNA levels in the substantia nigra of mice treated with MPTP, with or without 2‐DG (n = 3). (L–N) Behavioral assessments of MPTP‐induced mice with or without 2‐DG treatment: (L) time in the pole climbing test. (M) open‐field test showing representative images of mouse movement trajectories and total distance traveled. (N) Scores in the grasping test. n = 7 per group. Data are presented as mean ± SEM. ^*^ *p* < 0.05, ^**^ *p* < 0.01, and ^***^ *p* < 0.001. Statistical significance was determined by one‐way ANOVA or Student's t‐test as appropriate. ATPB, ATP synthase subunit beta. “n” refers to the number of mice per group. “N” refers to the number of biologically independent experiments.

We next evaluated the functional outcomes of this intervention. Systematic behavioral assessment revealed that 2‐DG administration ameliorated MPTP‐induced motor deficits, with increased exploratory activity in the open field test, improved coordination on the rotarod, and prolonged endurance in the hanging wire test (Figure 5L–N).

Together, these results indicate that the inhibition of H4K12la by 2‐DG rescues energy metabolism dysfunction, protects dopaminergic neurons, and improves motor function in PD models, thus positioning the H4K12la/HIF‐1α axis as a critical mediator of metabolic and neuronal vulnerability in PD.

### 3.6 HIF‐1α Regulates Mitochondrial Respiration and Energy Supply Through NDUFS1 in PD Cell Model

HIF‐1α serves as a master transcriptional regulator of metabolic reprogramming; it promotes the expression of glycolytic enzymes while simultaneously suppressing oxidative phosphorylation [32]. To investigate the mechanistic role of HIF‐1α in PD cell models, we first designed four siHIF‐1α RNA duplexes and validated their knockdown efficiency using western blot analysis. As shown in Figure S3, si4‐HIF‐1α (si‐4) significantly suppressed both HIF‐1α protein and *HIF‐1α* mRNA expression. This optimized siRNA (si‐4) was therefore used in all following mechanistic studies.

We then performed RNA‐seq analysis to investigate transcriptomic alterations following *HIF‐1α* knockdown in MPP^+^‐induced PD cell models. The heatmap analysis identified 466 differentially expressed genes (|log_2_FC| > 1, p < 0.05) in SN4741 cells transfected with siHIF‐1α upon treatment with MPP^+^ compared with MPP^+^‐treated controls; comprising 286 upregulated and 186 downregulated genes (Figure 6A). GO enrichment analysis revealed the significant association of these differentially expressed genes with mitochondrial respiratory chain complexes (Figure 6B). KEGG pathway analysis further demonstrated a prominent enrichment in oxidative phosphorylation (Figure 6C). Notably, the expression of NDUFS1, which encodes a core subunit of mitochondrial CI, was prominently altered (Figure 6D). Because HIF‐1α inhibits oxidative phosphorylation, we explored the regulation by HIF‐1α of NDUFS1. RT‐qPCR analysis indicated that, of the mitochondrial complex subunits that were assessed, the most significant transcriptional alterations occurred within CI components. Notably, NDUFS1 exhibited the most pronounced decrease in mRNA levels following MPP^+^ treatment (Figure 6E). This downregulation was further confirmed at the protein level using western blot analysis (Figure 6F).

**FIGURE 6:**
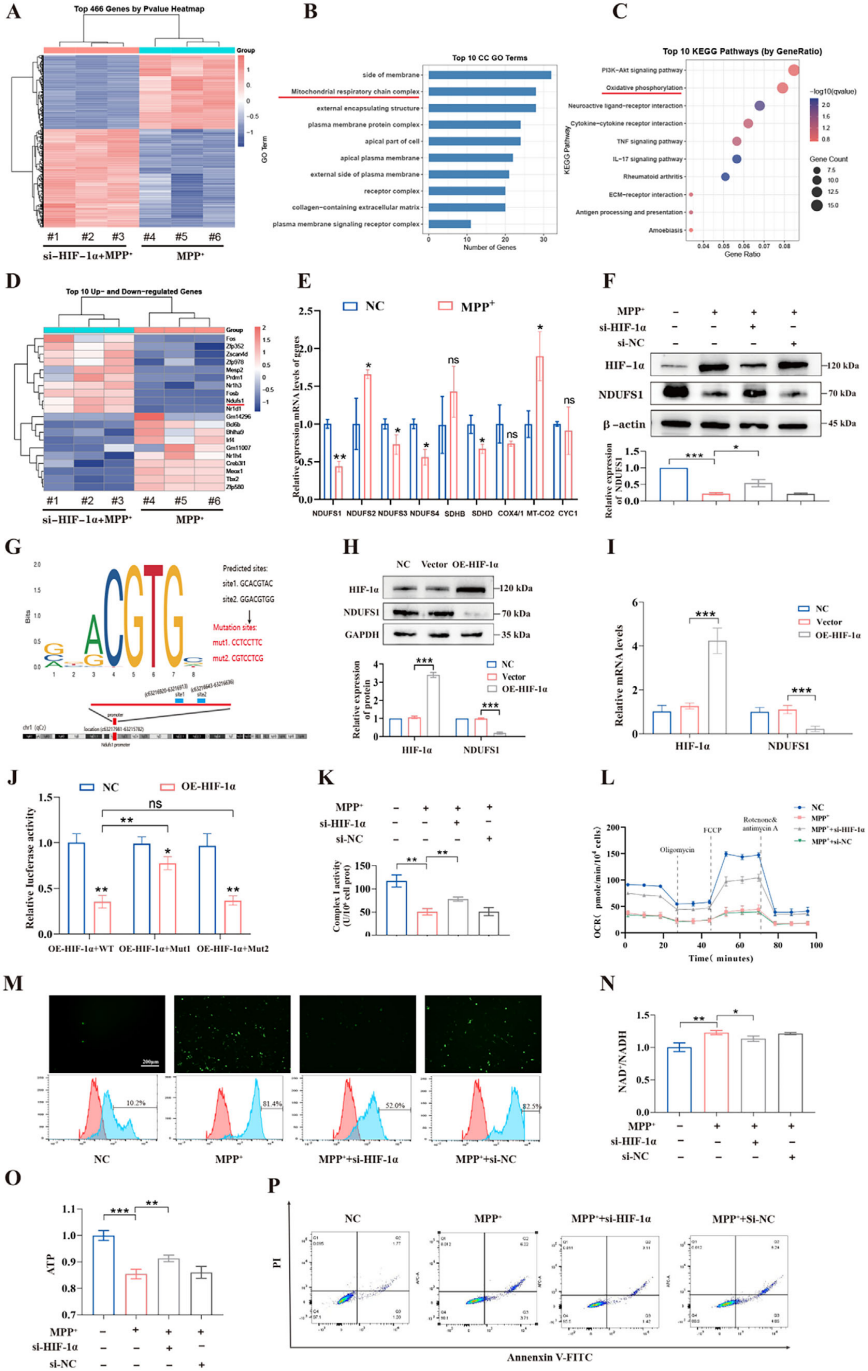
HIF‐1α transcriptionally represses NDUFS1 to impair mitochondrial Complex I function, leading to bioenergetic crisis and neuronal apoptosis. (A) The heatmap of RNA‐seq data showing differentially expressed genes in MPP^+^‐treated SN4741 cells upon HIF‐1α knockdown (si‐HIF‐1α) compared to MPP^+^ treatment alone (N = 3). (B) GO enrichment analysis of biological processes for the differentially expressed genes. (C) KEGG pathway enrichment analysis for the differentially expressed genes. (D) Heatmap showing top‐ranked differentially expressed genes from RNA‐seq data. (E) The mRNA levels of mitochondrial complex I core subunits, including *NDUFS1*, *NDUFS2*, and *NDUFS3*, in SN4741 cells treated with or without MPP^+^ were detected by RT‐qPCR. (F) Western blotting analysis of NDUFS1 protein levels in SN4741 cells under MPP^+^ treatment and/or HIF‐1α knockdown. (G) Schematic diagram of the WT and mutant (Mut1, Mut2) *NDUFS1* promoter reporter constructs, showing the predicted HIF‐1α binding sites. (H) Western blotting analysis of HIF‐1α and NDUFS1 protein levels in SN4741 cells transfected with HIF‐1α overexpression plasmid (OE‐HIF‐1α) or empty vector (N = 3). (I) RT‐qPCR analysis of *HIF‐1α* and *NDUFS1* mRNA levels in SN4741 cells from the experiment in (H). (J) Dual‐luciferase reporter assay in SN4741 cells co‐transfected with HIF‐1α overexpression plasmid and the indicated *NDUFS1* promoter constructs (N = 3). (K) CI enzymatic activity measured in SN4741 cells under MPP^+^ treatment and/or HIF‐1α knockdown (N = 3). (L) OCR profiles and derived parameters for mitochondrial function in SN4741 cells under MPP^+^ treatment and/or HIF‐1α knockdown (N = 3). (M) Intracellular ROS levels in SN4741 cells under MPP^+^ treatment and/or HIF‐1α knockdown (N = 3). (N,O) Measurements of (N) NAD^+^/NADH ratio and (O) ATP levels in SN4741 cells under MPP^+^ treatment and/or HIF‐1α knockdown (N = 3). (P) Apoptosis rate measured by flow cytometry in SN4741 cells under the indicated treatments (N = 3). Data are mean ± SEM. ns, not significant, ^*^ *p* < 0.05, ^**^ *p* < 0.01, and ^***^ *p* < 0.001. Statistical significance was determined by one‐way ANOVA or Student's t‐test as appropriate. NDUFS1, NADH: ubiquinone oxidoreductase core subunit S1; CI, Complex I; OCR, Oxygen Consumption Rate; ROS, Reactive Oxygen Species. “N” refers to the number of biologically independent experiments.

To evaluate whether HIF‐1α directly mediates this repression, we analyzed the *NDUFS1* promoter region and predicted two HIF‐1α‐binding elements using the JASPAR database of transcription factor binding motifs (Figure 6G). We then conducted gain‐ and loss‐of‐function experiments. Overexpression of HIF‐1α was sufficient to suppress *NDUFS1* mRNA and protein levels (Figure 6H,I). To establish direct transcriptional regulation, we performed dual‐luciferase reporter assays. HIF‐1α overexpression robustly repressed the activity of a wild‐type *NDUFS1* promoter construct. Crucially, this repression was almost completely abolished by the mutation of the first predicted hormone response element (HRE; Mut1), whereas mutation at Mut2 had no significant effect (Figure 6J). Together, these data indicate a direct mechanistic link: HIF‐1α binds to a specific HRE in the *NDUFS1* promoter to repress its transcription. This pathway may contribute to the suppression of oxidative phosphorylation in PD cell models, thus directly connecting HIF‐1α‐mediated metabolic reprogramming to mitochondrial dysfunction.

Having established that HIF‐1α transcriptionally repressed NDUFS1, we next investigated the functional and pathological consequences of this repression. As the largest core subunit of CI, NDUFS1 plays a critical role in maintaining CI structural stability and oxidative phosphorylation activity [33]. We first assessed CI enzymatic activity. As shown in Figure 6K, MPP^+^ treatment significantly reduced CI activity, and this impairment was partially rescued by HIF‐1α knockdown. CI dysfunction disrupts electron flow in the ETC, leading to electron leakage and ROS generation [34]. Consistent with this mechanism, evaluation of the oxygen consumption rate demonstrated that MPP^+^ treatment severely compromised respiration, proton leakage, and maximal respiration, leading to defective electron transfer through the mitochondrial ETC (Figure 6L). This dysfunction resulted in excessive ROS generation through aberrant oxygen reactions, ultimately causing NAD^+^ and ATP deficiency. Strikingly, HIF‐1α knockdown partially ameliorated MPP^+^‐induced maximal respiration, ROS accumulation, and the reduction of both NAD^+^ and ATP levels (Figure 6M–O). Given that apoptosis represents a terminal event consequent to neuronal energy depletion [35], we assessed the apoptotic rate using flow cytometry; HIF‐1α knockdown significantly attenuated MPP^+^‐induced apoptosis (Figure 6P).

Together, our findings suggest that HIF‐1α binds to specific sequences in the *NDUFS1* promoter and inhibits its expression, thereby suppressing mitochondrial respiration and disrupting energy supply, which ultimately leads to neuronal death.

### 3.7 Pharmacological Inhibition of HIF‐1α Increased NDUFS1 Expression, Prevents Dopaminergic Neuron Loss, and Improves Motor Function in PD Mice

To investigate whether the pharmacological inhibition of HIF‐1α can replicate our genetic findings and exert therapeutic effects, we administered the brain‐penetrant HIF‐1α inhibitor PX‐478 to MPTP‐induced PD mice [36]. First, a dose–response experiment confirmed that PX‐478 (25, 50, and 100 mg/kg) dose‐dependently suppressed HIF‐1α expression in the substantia nigra and striatum (Figure 7A,B). On the basis of its maximal inhibitory effect, the highest dose (100 mg/kg) was selected for all subsequent studies. At this dose, PX‐478 effectively reversed the molecular pathology of the PD model. Western blot analysis revealed that PX‐478 co‐treatment significantly reduced the MPTP‐induced elevation of HIF‐1α. Conversely, it partially restored the suppressed expression of its downstream target NDUFS1 in both brain regions (Figure 7C,D). Next, we investigated whether this restoration of NDUFS1 protein led to the recovery of mitochondrial CI function. Using a substrate‐specific assay, we observed that MPTP treatment significantly reduced CI enzymatic activity in the substantia nigra and striatum, and this impairment was partially but significantly rescued by PX‐478 administration (Figure 7E,F).

**FIGURE 7:**
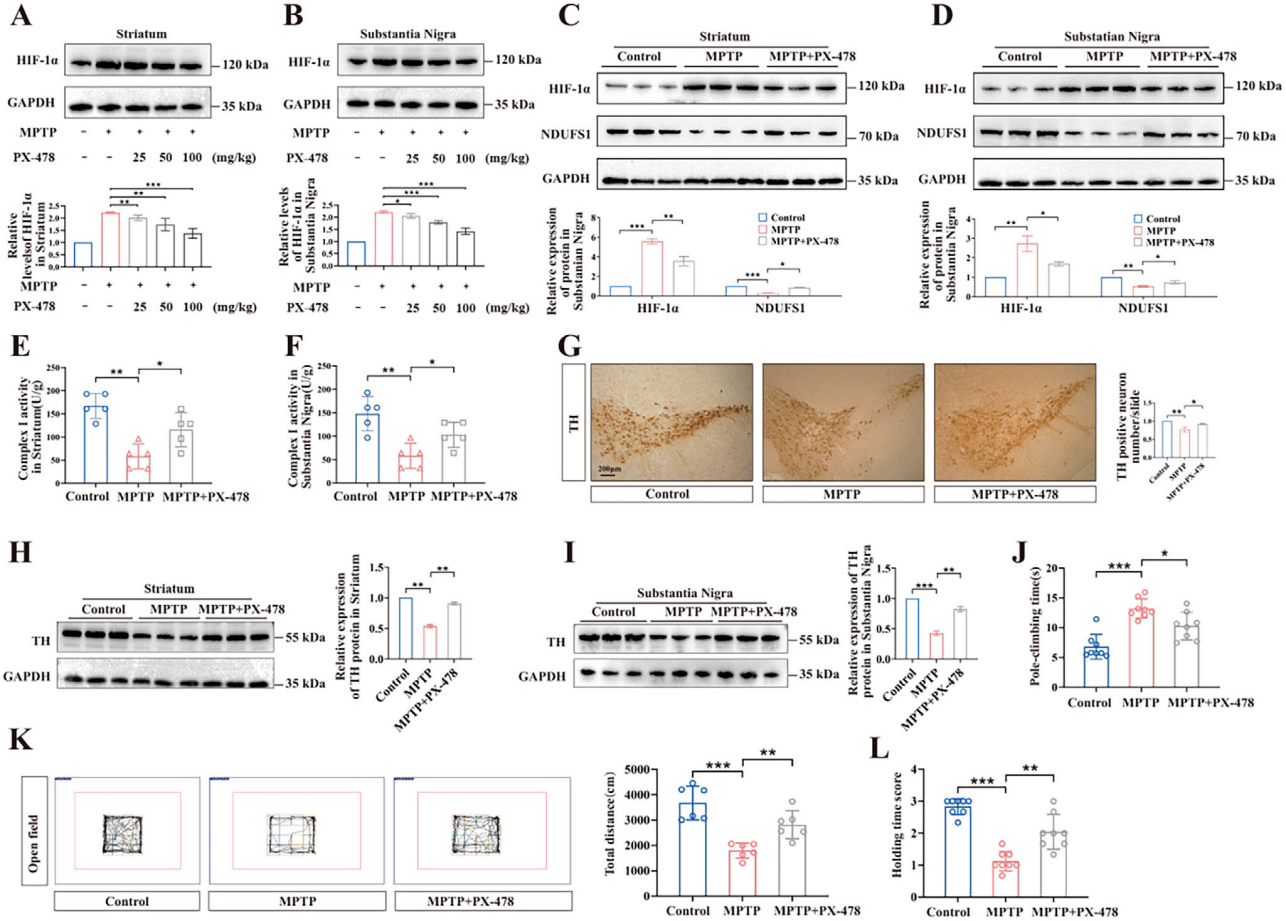
Pharmacological inhibition of HIF‐1α by PX‐478 restores NDUFS1 expression, ameliorates neuropathology, and improves motor function in MPTP‐induced PD mice. (A,B) Western blotting analysis and quantification of HIF‐1α protein levels in the (A) striatum and (B) substantia nigra of MPTP‐induced PD mice treated with low (25 mg/kg), medium (50 mg/kg), or high (100 mg/kg) doses of PX‐478 (n = 3). (C,D) Western blotting analysis and quantification of HIF‐1α and NDUFS1 protein levels in the (C) striatum and (D) substantia nigra of mice treated with MPTP and/or PX‐478 (n = 3). (E,F) CI enzymatic activity measured in the (E) striatum and (F) substantia nigra of mice treated with MPTP and/or PX‐478 (n = 3). (G) Representative immunohistochemical images and quantitative analysis of TH expression in the midbrain of mice treated with MPTP and/or PX‐478 (n = 3). Scale bar, 200 µm. (H,I) Western blot analysis and quantification of TH protein levels in the (H) striatum and (I) substantia nigra of mice treated with MPTP and/or PX‐478 (n = 3). (J–L) Behavioral assessments of MPTP‐induced mice with or without PX‐478 treatment: (J) time in the pole climbing test; (K) open‐field test showing representative images of mouse movement trajectories and total distances traveled; (L) scores in the grasping test. n = 8 per group. Data are presented as mean ± SEM. ^*^ *p* < 0.05, ^**^ *p* < 0.01, and ^***^ *p* < 0.001. Statistical significance was determined by one‐way ANOVA or Student's t‐test as appropriate. PX‐478, S‐2‐amino‐3‐[4'‐N,N‐bis (2‐chloroethyl) amino]phenyl propionic acid N‐oxide dihydrochloride. “n” refers to the number of mice.

This molecular and functional rescue translated into neuroprotection. Consistent with the recovery of NDUFS1, PX‐478 treatment also partially restored the protein levels of TH in the substantia nigra and striatum of MPTP mice (Figure 7G–I). Ultimately, this neuroprotection led to significant functional improvement. Quantitative behavioral analyses demonstrated that PX‐478 treatment markedly attenuated MPTP‐induced motor deficits; specifically, it enhanced exploratory activity, improved coordination, and increased endurance (Figure 7J–L). Collectively, these results indicate that the pharmacological inhibition of HIF‐1α with PX‐478 rescues the NDUFS1/CI axis, thereby mitigating dopaminergic neurodegeneration and motor dysfunction in a PD mouse model.

### 3.8 P300 Drives the Lactylation of H4K12 in the PD Cell Model

Lysine lactylation can occur through non‐enzymatic reactions under high lactate concentrations, or via enzymatic regulation involving dedicated “writer” and “eraser” proteins [8, 9]. Given the mechanistic parallels between lactylation and acetylation, we hypothesized that known histone acetyltransferases (P300/CBP, GCN5, HBO1, KAT2B, histone acetyltransferase KAT5/TIP60, histone acetyltransferase KAT6A/MOZ, and histone acetyltransferase KAT6B/MORF) might also catalyze H4K12la [37]. To identify potential regulators, we systematically screened the established acetyltransferase writers in PD cell models. Among the candidates, P300 emerged as a key regulator; both its mRNA and protein levels were significantly upregulated following MPP^+^ treatment (Figure 8A,B). We therefore investigated whether P300 directly interacts with H4K12la. Co‐immunoprecipitation experiments demonstrated reciprocal binding between P300 and H4K12la in PD cell models; P300 antibodies effectively precipitated H4K12la‐modified histones and vice versa (Figure 8C). Furthermore, immunofluorescence co‐localization analysis demonstrated the clear nuclear co‐localization of H4K12la and P300 (Figure 8D and E). To further analyze the mechanisms of interaction between P300 and H4K12la, we performed molecular docking predictions and constructed an interaction model using the STRING database (https://cn.string‐db.org/). The prediction results indicated the presence of six covalent hydrogen bonds between P300 and H4K12la, which suggest favorable molecular docking characteristics (Figure 8F). Together, these data suggest that P300 is a direct binding partner of H4K12la.

**FIGURE 8:**
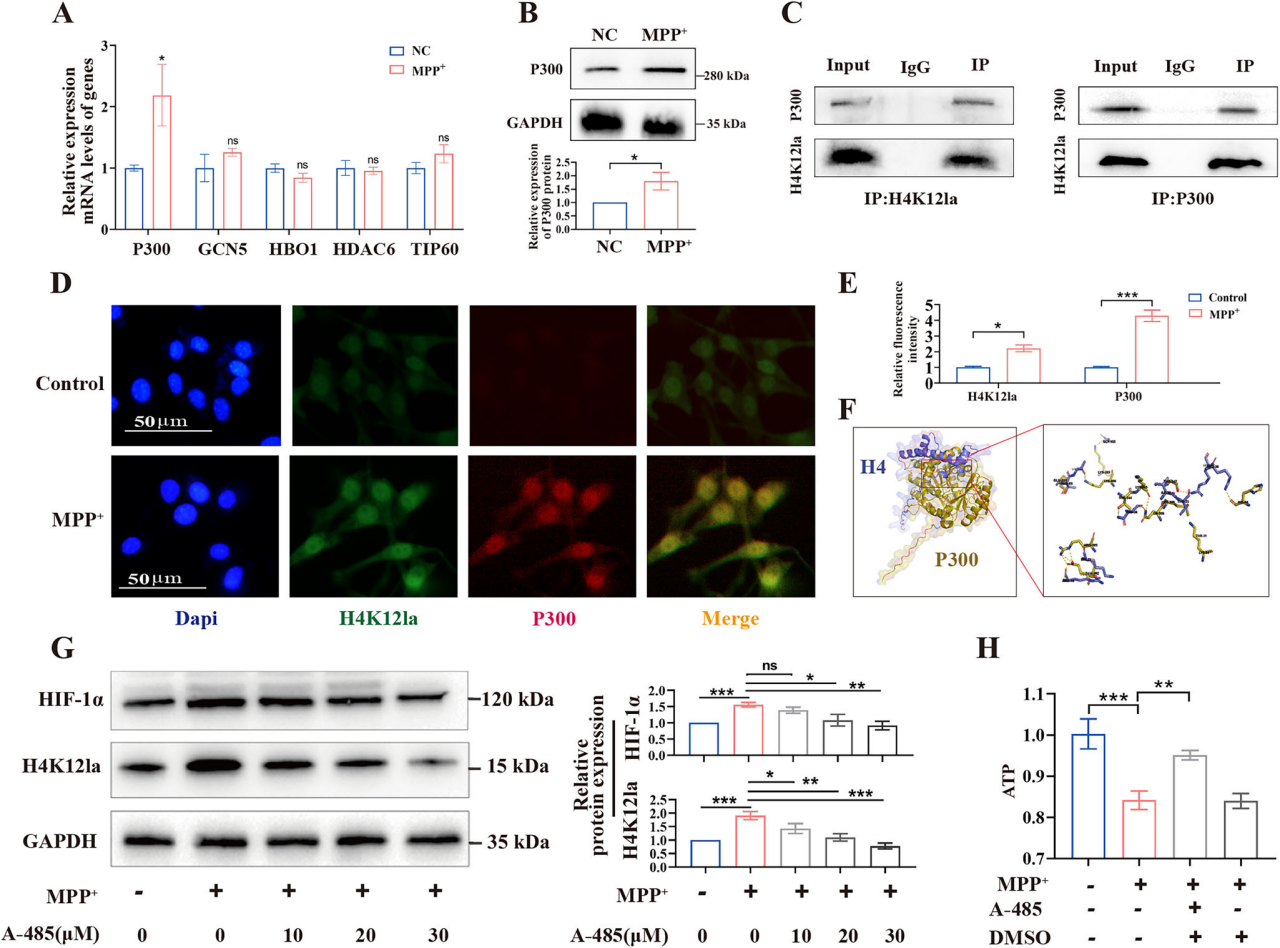
P300 interacts with and regulates H4K12la to modulate cellular energy metabolism in PD models. (A) RT‐qPCR analysis of P300, GCN5, HBO1, HDAC6, TIP60 mRNA expression in SN4741 cells treated with or without MPP^+^ (N = 3). (B) Western blotting analysis and quantification of P300 protein levels in SN4741 cells treated with or without MPP^+^ (N = 3). (C) Co‐IP assays showing reciprocal binding between P300 and H4K12la in MPP^+^‐treated SN4741 cells (N = 3). (D) Representative immunofluorescence images showing the co‐localization of P300 (red) and H4K12la (green) in the nucleus of SN4741 cells, and (E) the quantitative analysis of co‐localization. DAPI (blue) stains nuclei (N = 3). Scale bar, 50 µm. (F) Predicted molecular docking model of the interaction between P300 and H4K12la, generated using the STRING database, indicating the presence of six hydrogen bonds. (G) Western blotting analysis of H4K12la and HIF‐1α protein levels in SN4741 cells treated with MPP^+^ in the presence or absence of the indicated concentrations of A‐485 (N = 3). (H) Intracellular ATP levels in SN4741 cells treated with MPP^+^ and/or A‐485 (N = 3). Data are presented as mean ± SEM. ns, not significant, ^*^ *p* < 0.05, ^**^ *p* < 0.01, and ^***^ *p* < 0.001. Statistical significance was determined by one‐way ANOVA or Student's t‐test as appropriate. “N” refers to the number of biologically independent experiments.

To determine whether the enzymatic activity of P300 is required for H4K12la maintenance and downstream signaling, we used the selective P300 inhibitor A‐485. A‐485 is a CoA competitive catalytic inhibitor of P300, and L‐lactyl‐CoA is indispensable for lactylation [38]. Treatment with A‐485 markedly diminished H4K12la levels and concurrently reduced the expression of HIF‐1α, which is a key transcriptional target of H4K12la (Figure 8G). Functionally, this inhibition partially rescued the MPP^+^‐induced deficit in ATP production (Figure 8H). Collectively, our findings indicate that the histone acetyltransferase P300 is a critical driver of H4K12la in PD cell models. Specifically, our results suggest that P300 binds to H4K12la, and its catalytic activity is necessary for sustaining H4K12la levels, thereby regulating downstream HIF‐1α expression and modulating cellular energy metabolism.

## 4 Discussion

To our knowledge, this is the first study to demonstrate that lactate levels and H4K12la are significantly elevated in both PD patients and dopaminergic neurons exposed to MPP^+^/MPTP, thus suggesting a potential role of lactate‐driven histone lactylation in PD pathogenesis. Our findings indicate that H4K12la is specifically upregulated in PD models. Moreover, through integrated genome‐wide transcriptional profiling, RNA‐seq, and co‐immunoprecipitation results, we were able to delineate a complete pathogenic axis: the histone acetyltransferase P300 drives H4K12la, which in turn activates the transcription factor HIF‐1α to suppress the expression of the mitochondrial CI subunit NDUFS1. This cascade mechanistically links aberrant histone lactylation to the energy metabolism deficits central to PD.

The early diagnosis and effective treatment of PD remain critical challenges to overcome. Thus, the identification of early biomarkers and specific therapeutic targets is of great importance. In 1989, Schapira et al. first reported a 30%–40% reduction in mitochondrial CI activity in the substantia nigra of PD patients [39]. Subsequent studies observed decreased CI activity even in PD patients with Braak stages I–II, which suggests that mitochondrial dysfunction is an early event in PD pathogenesis [40]. Lactate is primarily derived from glycolysis; it accumulates in the brain when oxidative phosphorylation is impaired in dopaminergic neurons, thus forcing cells to rely more heavily on glycolysis for ATP production [41]. Notably, elevated lactate levels have been detected in the substantia nigra pars compacta of MPTP‐induced PD mouse models and in MPP^+^‐treated SH‐SY5Y cells. HK2 inhibitors or *Hk2* siRNA effectively reduce lactate production, attenuates dopaminergic neuron apoptosis, and improves motor deficits in mice, thereby implicating lactate as a key player in PD pathology [42]. However, the precise mechanistic role of lactate in PD progression remains poorly understood. Following the groundbreaking discovery by Zhao et al. that lactate directly mediates histone lactylation on lysine residues, histone lactylation has emerged as a metabolic sensor that links cellular energy status to epigenetic regulation [8]. Recent studies have demonstrated that protein lactylation modulates neuroinflammation, neuronal excitability, and neurodevelopment, playing a crucial epigenetic role in neurological disorders such as stroke, Alzheimer's disease, and hypoxic‐ischemic encephalopathy [3, 43]. Building on these findings, we aimed to define the precise role of lactate and its epigenetic derivative, histone lactylation, in PD pathogenesis and to evaluate their potential as disease markers and therapeutic targets.

We identified that resting plasma lactate levels were elevated in PD patients, indicating systemic metabolic dysregulation. Furthermore, MRS analysis revealed regional lactate accumulation in the PD striatum. Notably, global protein lactylation in peripheral blood mononuclear cells was significantly increased in PD patients, thereby establishing it as a novel molecular feature of PD. Moreover, the observation of impaired post‐exercise lactate clearance in PD patients supports the notion of underlying mitochondrial dysfunction. Importantly, the lack of correlation with disease severity suggests that lactylation dysregulation may be an early‐onset and persistent event in PD pathogenesis.

There is clear cell‐type heterogeneity in the functions of histone lactylation in the nervous system. For example, in Alzheimer's disease, H4K12la in microglia drives transcription of glycolytic genes, forming a pro‐inflammatory positive feedback loop [15]. By contrast, in stroke models, lactylation primarily regulates neuronal damage and repair processes [44]. The present study focused on dopaminergic neurons—the central vulnerable cell type in PD—and demonstrated for the first time that, among various histone lactylation modifications, H4K12la shows the most pronounced and specific upregulation in dopaminergic neurons. This finding suggests that the H4K12 site may serve as a key epigenetic sensor in these neurons in response to metabolic stress, such as lactate accumulation. Moreover, our results indicate that H4K12la directly transcriptionally activates HIF‐1α, which is a master metabolic regulator. Subsequently, HIF‐1α binds to the promoter of *NDUFS1*, which encodes the core subunit of mitochondrial CI, and represses its expression. To further validate this, we introduced an H4K12R mutation into SN4741 cells. This mutation impaired MPP^+^‐induced HIF‐1α upregulation, confirming H4K12la as an upstream signal for HIF‐1α activation. Given that CI deficiency is a hallmark of PD, this suppression may create a vicious cycle: impaired oxidative phosphorylation leads to energy depletion (i.e., reduced ATP/NAD^+^) and oxidative stress (i.e., elevated ROS), thus collectively driving neuronal degeneration. The therapeutic potential of this axis was confirmed by the efficacy of 2‐DG and PX‐478, which target upstream nodes and rescue NDUFS1 expression and motor function in vivo.

The rate of lactylation primarily depends on the level of lactyl‐CoA and the activity of enzymes that transfer the lactyl group from lactyl‐CoA to histones [8]. The acetyltransferase P300/CBP is a key catalytic factor for histone lactylation across various cell types [45, 46]. Our results suggest that P300 can induce histone lactylation and plays a role in regulating gene transcription in dopaminergic neurons. Using a specific inhibitor to block P300 significantly reduced histone lactylation levels, thereby alleviating mitochondrial energy supply deficits. These results support the idea that histone lactylation is involved in mitochondrial energy impairment and PD development. Furthermore, we noted that P300 protein expression increased after MPP^+^ stimulation, which indicates that elevated intracellular lactate levels and increased P300 expression may jointly enhance histone lactylation in PD.

However, several limitations of the present study should be considered. First, although MPTP/MPP^+^ models are valuable for studying acute mitochondrial dysfunction, they may not fully recapitulate the progressive α‐synuclein accumulation observed in human PD. Second, although our H4K12R mutagenesis in SN4741 cells supports a causal role of H4K12la in HIF‐1α activation, in vivo validation remains to be explored. Third, H4K12la emerged as the dominant modification; however, its potential interplay with other lactylation sites and competing post‐translational modifications (e.g., acetylation) at shared lysine residues remains unresolved. Finally, although 2‐DG and PX‐478 demonstrated therapeutic efficacy, their broad‐spectrum effects highlight the need for the development of H4K12la‐specific modulators.

In conclusion, our work defined the P300/H4K12la/HIF‐1α/NDUFS1 axis as a pathogenic cascade in PD. H4K12la emerged as a crucial epigenetic link between lactate metabolism and mitochondrial failure. This pathway may offer a novel diagnostic framework and promising therapeutic targets, as evidenced by the efficacy of its pharmacological inhibition in PD models.

## Author Contributions

Y.X. organized and designed the project. J.L., Y.L., H.H., Y.B., Q.O., W.F., Y.T. and X.Q. performed and validated the experiments. W.H., F.L., Y.L. and X.L. performed the experiments, analyzed data, generated figures, and drafted the preliminary manuscript. L.G., Y.X., Y.S., and J.W. critically edited and reviewed the manuscript.

## Conflicts of Interest

The authors declare no conflicts of interest.

## Supporting information




**Supporting File**: advs78220‐sup‐0001‐SuppMat.docx.

## Data Availability

Access to the datasets supporting this research can be obtained from the corresponding author, contingent upon the submission of a reasonable request.
