# Supplementary material for: H4K12 Lactylation Impairs Dopaminergic Neuron Energy Metabolism via the HIF‐1α/NDUFS1 Pathway in Parkinson's Disease
Source: Adv Sci (Weinh). 2026 Oct 8:e78220. Online ahead of print. doi: 10.1002/advs.78220 (PMC13650024; doi:10.1002/advs.78220)
Supplement: Supplementary file 1 [file ADVS-9999-e78220-s001.docx]

**Supplementary Materials for**

**H4K12 Lactylation Impairs Dopaminergic Neuron Energy Metabolism via the HIF-1α/NDUFS1 Pathway in Parkinson’s Disease**

*Yingyao Liu ^1,#^, Xi Li ^1,#^, Wenxuan He ^1,#^, Fenglin Li ^1^, Qican Ou ^2^, Yao Liu ^1^, Jiang Lei ^3^, Yanzi Bao ^1^, Huaxin Huang ^1^, Wei Feng ^1^, Yuanguang Tang ^1^, Xiaohui Qiu ^1^, Lu Gan ^1^, Yuefei Shen ^1^, Jin Wang ^1^, Yousheng Xiao* *^1,*^*

^1^Department of Neurology, the First Affiliated Hospital of Guangxi Medical University, Nanning, China.

^2^Department of Neurology, Wuming Hospital Affiliated to Guangxi Medical University, Nanning, China.

^3^Department of Neurology, Guangxi Hospital Division of The First Affitiated Hospital, Sun Yat-sen University, Nanning, China.

*Correspondence to: Yousheng Xiao, [xys135@126.com](mailto:xys135@126.com).

#The authors contributed equally to this work.

**This file includes:**

**Supplementary Figures and Figure Legends**

**Supplementary Table S1-S5**

**Supplementary Figure Legends**

**Supplementary Figure 1. Upregulation of HK2 and LDHA in MPTP-induced PD mice.** (A and B) Western blot analysis and quantification of HK2 protein levels in the (A) striatum and (B) substantia nigra of MPTP-induced PD mice (n=3). (C and D) Western blot analysis and quantification of LDHA protein levels in the (C) striatum and (D) substantia nigra of MPTP-induced PD mice (n=3). (E and F) Representative immunofluorescence images of midbrain sections showing a marked decrease in TH signal (green) and a concurrent increase in HK2 signal (red) in MPTP-treated mice. Scale bar, left, 200 μm; right, 20 μm. (n=3). (G and H) Representative immunofluorescence images ofmidbrain sections showing a marked decrease in TH signal (green) and a concurrent increase in LDHA signal (red) in MPTP-treated mice. Scale bar, left, 200 μm; right, 20 μm. (n=3). (I) The effect of different concentrations of MPP^+^ on cell viability was detected using the CCK-8 kit (N=3) . Data are mean ± SEM. *p < 0.05, **p < 0.01, and **p < 0.001. Statistical significance was determined by one-way ANOVA or Student's t-test as appropriate. TH, Tyrosine Hydroxylase; HK2, Hexokinase 2; LDHA, Lactate Dehydrogenase A; "n" refers to the number of mice; "N" refers to the number of independent cell culture preparations.

**Supplementary Figure 2. Histone H4K12 lactylation regulates HIF-1α expression in MPP^+^-treated SN4741 cells and MPTP-induced PD mice.** (A) RT-qPCR analysis of *HIF-1α* mRNA cxpression in SN4741 cells treated with 0.5mM MPP^+^, NaLa, Lac, or NaCI for 24 hours (N=3). (B) Sanger sequencing chromatograms confirming the H4K12R point mutation (Upper: H4-WT; Lower: H4-K12R). (C)SN4741 cells transfected with H4-WT or H4-K12R were treated with or without lactate, and H4K12la levels were detected by Western blotting (N=3). (D, E) HIF-1α protein (D) and mRNA (E) levels in cells treated as in (C) (N=3). (F) *HIF-1α* mRNA levels were measured by RT-qPCR in cells transfected with vector, H4-WT, or H4-K12R and treated with or without MPP^+^ (N=3). (G) The expression levels of multiple histone lactylation sites (H4K121a, H3K9la, H3K14la, H3K181a, and H3K231a) in MPP^+^-treated SN4741 cells with or without 2-DG co-treatment were detected by Western blotting (N=3). (H) RT-qPCR analysis of *HIF-1α* mRNA expression in MPP^+^-treated SN4741 cells with or without 2-DG co-treatment (N=3). (I) Quantification of relative fluores-cence intensity shown in Figure 4R (n=3). (J, K) Representative immunofluorescence images showing the co-localization of H4K12la (green) and HIF-1α (red) in the substantia nigra of control, MPTP, and MPTP+2DG mice. Scale bars: left, 200 μm; right, 50 μm. Data are resented as mean ± SEM. ns, no significance,*p <0.05, **p <0.01, and ***p<0.001. Statistical significance was determined by one-way ANOVA or Student's t-test as appropriate. "n" refers to the number ofmice. "N" refers to the number of independent cell culture preparations.

**Supplementary Figure 3.** **HIF-1α knockdown efficiency of four siRNAs.** Western blotting analysis of the interference efficiency of four si-HIF-1α RNA duplexes (N=3, meaning three independent experiments). Data are mean ± SEM. ns, no significance, *p < 0.05 and **p < 0.01. Statistical significance was determined by one-way ANOVA.

**Supplementary Table 1.** Demographic and clinical characteristics of Parkinson's Disease and healthy controls.

| **Variables** | **PD(n=83)** | **Healthy control(n=83)** | **Comparison** |
| --- | --- | --- | --- |
| Gender (male/female) | 48/35 | 46/37 | χ² = 0.10, df = 1, p = 0.75 |
| Age (years) | 62.0±11.93 | 61.5±12.73 | t = 0.26, df = 164, p = 0.79 |
| Age of onset (years) | 58.07±11.75 |  |  |
| Disease duration (years) | 3.99±3.466 |  |  |
| Hoehn and Yahr stage (number per stage 1/1.5/2/2.5/3/4/5) | 7/3/27/13/21/10/2 |  |  |
| Quiescent Blood lactate (mmol/L) | 2.68±0.66 | 2.24±0.61 | t = 4.33, df = 164, p < 0.0001 |
| Exercise Blood lactate (mmol/L) | 3.10±1.06 (n=31) | 3.76±0.91 (n=31) | t = 2.617, df = 60, p = 0.011 |
| Rest15min Blood lactate (mmol/L) | 2.80±0.90 (n=31) | 3.30±0.81 (n=31) | t = 2.302, df = 60, p = 0.024 |
| UPDRS III score | 36.11±17.36 |  |  |
| LED (mg) | 436.1±189.9 |  |  |

Note: Data are mean ± SD unless specified otherwise.

Note: “n” refers to the number of participants.

Abbreviations: PD, Parkinson's disease; UPDRS, unified Parkinson's disease rating scale; LED, Levodopa Equivalent Dose.

**Supplementary Table 2.** Demographic and clinical characteristics of Parkinson's Disease and healthy controls.

| Striatal lactate peak | PD (n=36) | Healthy control(n=13) | Total(n=49) | p-value |
| --- | --- | --- | --- | --- |
| Present, n(%) | 21(58.3） | 3(23.1） | 24(49.0) | 0.029* |
| Absent, n(%) | 15(41.7） | 10(76.9) | 25(51.0) |  |

*Fisher’s exact test

**Supplementary Table 3.** The siRNA HIF-1α sequence.

| Genes | Primer sequence (5'→3') |
| --- | --- |
| Si-1 | S: GGAGGACGAUGAACAUCAATT |
|  | AS: UUGAUGUUCAUCGUCCUCCTT |
| Si-2 | S: GCAUUGUGUGUGUGAAUUATT |
|  | AS: UAAUUCACACACACAAUGCTT |
| Si-3 | S: GUUACGAUUGUGAAGUUAATT |
|  | AS: UAACUGGAAAUCAUCAUCCTT |
| Si-4 | S: GUUCUAACCCAUUGUGGA |
|  | AS: UUAACUUCACAAUCGUAACTT |
| Si-NC | S: UUCUCCGAACGUGUCACGUTT |
|  | AS: ACGUGACACGUUCGGAGAATT |

Mus, Mus musculus; S, sense; AS, antisense.

**Supplementary Table 4.** Real-time quantitative PCR primer sequences.

| Genes | Primer sequence (5'→3') |
| --- | --- |
| HIF-1α | F: AGCCCTCCAAGTATGAGCAC |
|  | R: TGCCTTAGCAGTGGTCGTTT |
| GAPDH | F: GGGAGCCAAAAGGGTCAT |
|  | R: GAGTCCTTCCACGATACCAA |
| TH | F：CCAAGGTTCATTGGACGGC |
|  | R：CTCTCCTCGAATACCACAGCC |
| NDUFS1 | F: TGCAAATCCCTCGATTCTGTTAC |
|  | R: GCTTTCTCAATCTCTACCAGGC |
| NDUFS2 | F: GCTGTTATGTACCCAAGCAAAGA |
|  | R: TCCCCACTCAATTCCATCACT |
| NDUFS3 | F: TGTGGCTGAAATCTTGCCCAA |
|  | R: AGTTGAAGCGCAGAGACAACA |
| NDUFS4 | F: TGCTCGCAATAACATGCAGTC |
|  | R: GATCAGCCGTTGATGCCCAA |
| SDHB | F: ACCTTCCGAAGATCATGCAGA |
|  | R: GTGCAAGCTAGAGTGTTGCCT |
| SDHD | F: TTGCTCTGCGATGGACTATTCC |
|  | R: CAAGGCATCCCCATGAACAT |
| COX4I1 | F: GAGAAAGTCGAGTTGTATCGCA |
|  | R: GCTTCTGCCACATGATAACGA |
| MTCO2 | F: AGACGCTACTTCCCCTATCA |
|  | R: GGTCGTGTAGCGGTGAAAGT |
| CYC1 | F: CAGCTTCCATTGCGGACAC |
|  | R: GGCACTCACGGCAGAATGAA |
| P300 | F: ACCCTGTCATGCAAAGCCT |
|  | R: TGTCGGCAAGAAGGCCATAC |
| GCN5 | F: CAGTGGTGGAGGGGTCTCTA |
|  | R: AAACATTGTCTGGCGCTCTC |
| HBO1 | F: ATTCTGGACTGAGCAAAGAACAG |
|  | R: GTCATACTCGCTTGTCAGGTTTT |
| HDAC6 | F:TCCACCGGCCAAGATTCTTC |
|  | R: GCCTTTCTTCTTTACCTCCGCT |
| TIP60 | F: ATGTGTGCTACCGTCTCCTA |
|  | R: CTCGATCCTCCGGGAAGTACT |
| HIF-1α promoter | F: CCATAGCTCCCAGCATAGCC |
|  | R: TCCGTCTTGCCAAACCTCTC |

Mus, Mus musculus; F, forward; R, reverse; GAPDH

**Supplementary Table 5.** Genomic locations and base substitutions used to construct the NDUFS1 promoter mutant vectors (Mut1 and Mut2) for dual-luciferase reporter assays.

|  | Gene location | Gene sequence  (Predicted sites→Mutation sites) |
| --- | --- | --- |
| *NDUFS1* promoter-Mut1 | c63216920-63216913 | GCACGTAC→CCTCCTTC |
| *NDUFS2* promoter-Mut2 | c63216943-63216936 | GGACGTGG→CGTCCTCG |

The table details the genomic locations and base substitutions applied to construct the

mutant vectors (Mut1 and Mut2) for dual-luciferase assays. The mutations were introduced to validate the direct transcriptional regulation of NDUFS1 by HIF-1α. Mus,

Mus musculus.
